# Supplementary material for: Functional rewiring of G protein-coupled receptor signaling in human labor
Source: Cell Rep. 2022 Sep 6;40(10):111318. doi: 10.1016/j.celrep.2022.111318 (PMC9638024; doi:10.1016/j.celrep.2022.111318)
Supplement: Document S1. Figures S1–S6 and Table S1 [file mmc1.pdf]

Cell Reports, Volume *40*

Supplemental information

Functional rewiring of G protein-coupled receptor signaling in human labor

Abigail R. Walker, Camilla B. Larsen, Samit Kundu, Christina Stavrinidis, Sung Hye Kim, Asuka Inoue, David F. Woodward, Yun S. Lee, Roberta Migale, David A. MacIntyre, Vasso Terzidou, Francesca Fanelli, Shirin Khanjani, Phillip R. Bennett, and Aylin C. Hanyaloglu

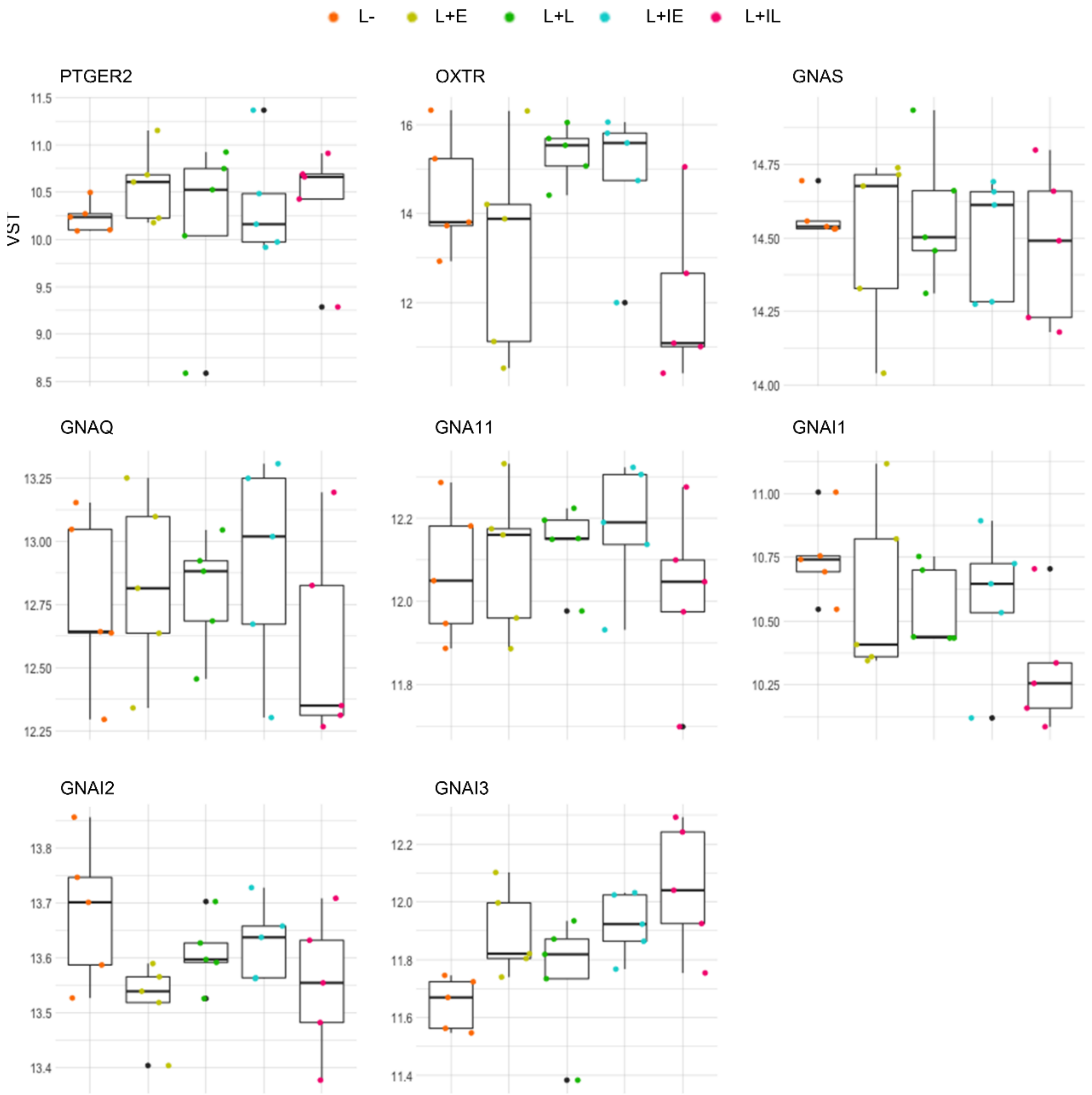

Figure S1. Expression of EP2, OTR and their Ga subunits in myometrial cells during labor, related to Figure 1. Expression levels of EP2 (Ptger2), OTR (Oxtr) and Gα subunits associated with EP2 and OTR signaling, GNAS, GNAI1/I2/I3/O and GNAQ/11, in term pregnant myometrium via RNA-Seq of patient samples used for functional studies in Fig. 1A-C. L- = non-laboring, L+E = early-stage spontaneous labour, L+L= late-stage spontaneous labour, L+IE =

early-stage induced labour, L+IL= late-stage induced labor. Early labor refers to cervical dilation < 3 cm, late labour refers to cervical dilation > 3 cm. Each circle represents one patient with outliers indicated with black circle. VST = variance stabilizing transformation. n=5.

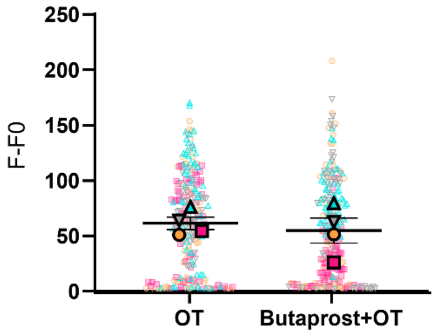

Figure S2. Pre-treatment of myocytes with butaprost does not affect OT-induced intracellular Ca^{2+} responses, related to Figure 2. Intracellular Ca^{2+} release in cultured term pregnant myocytes. Cells were imaged live before and following stimulation with butaprost (10 µM), with or without 1h OT pretreatment (100 nM). Data is the maximal fluorescent intensity normalized to unstimulated baseline (F-F0) shown for each cell analyzed, overlaid with the mean of the maximum cell intensity per experiment ± SEM. Each cell analyzed is represented and color coded for each biological repeat. Data shown as mean intensity ± SEM. 30 cells were imaged in duplicate per sample, n=4.

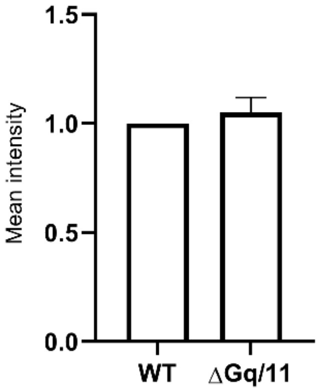

Figure S3. Cell surface expression of FLAG-tagged EP2 in ΔGαq/11 versus wildtype (WT) HEK 293 cells, related to Figure 4. The cell surface levels of human FLAG-tagged EP2 transiently expressed in either HEK 293 cells lacking GNAQ and GNA11 (ΔGαq/11) or WT HEK 293 cell was analyzed by anti-FLAG antibody staining in live cells and quantitated by flow cytometry. Mean fluorescence intensity is normalized to WT expression. Data are mean ± SEM of n=3.

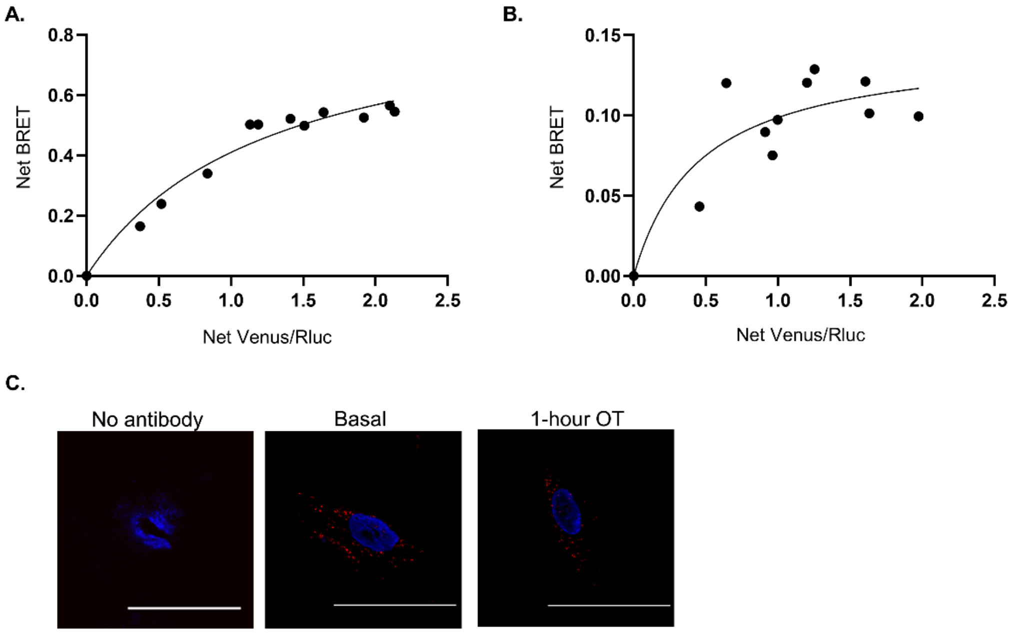

Figure S4. EP2-OTR heteromers are pre-formed in both HEK cells and primary human term pregnant myometrial cells, related to Figure 5. (A-B) Bioluminescence resonance energy transfer (BRET) saturation curves in (A) HEK 293 cells and (B) primary non-laboring myometrial cells, transiently transfected with EP2-RLuc8 and increasing levels of OTR-Venus. Coelenterazine was added at a final concentration of 5 µM. Emission was measured at 488nm and 530nm. Venus YFP was measured via excitation at 485nm and emission at 540nm. Representative curve shown, n=3. **(C)** Proximity ligation assay (PLA) with and without OT treatment (100 nM, 1 h) in primary myocytes to detect EP2/OTR heteromers. Red spots indicate positive PLA signal. DAPI for nuclear staining is in blue. Representative images from ~20 cells imaged across 2 patient samples. Scale bar = 50 µm.

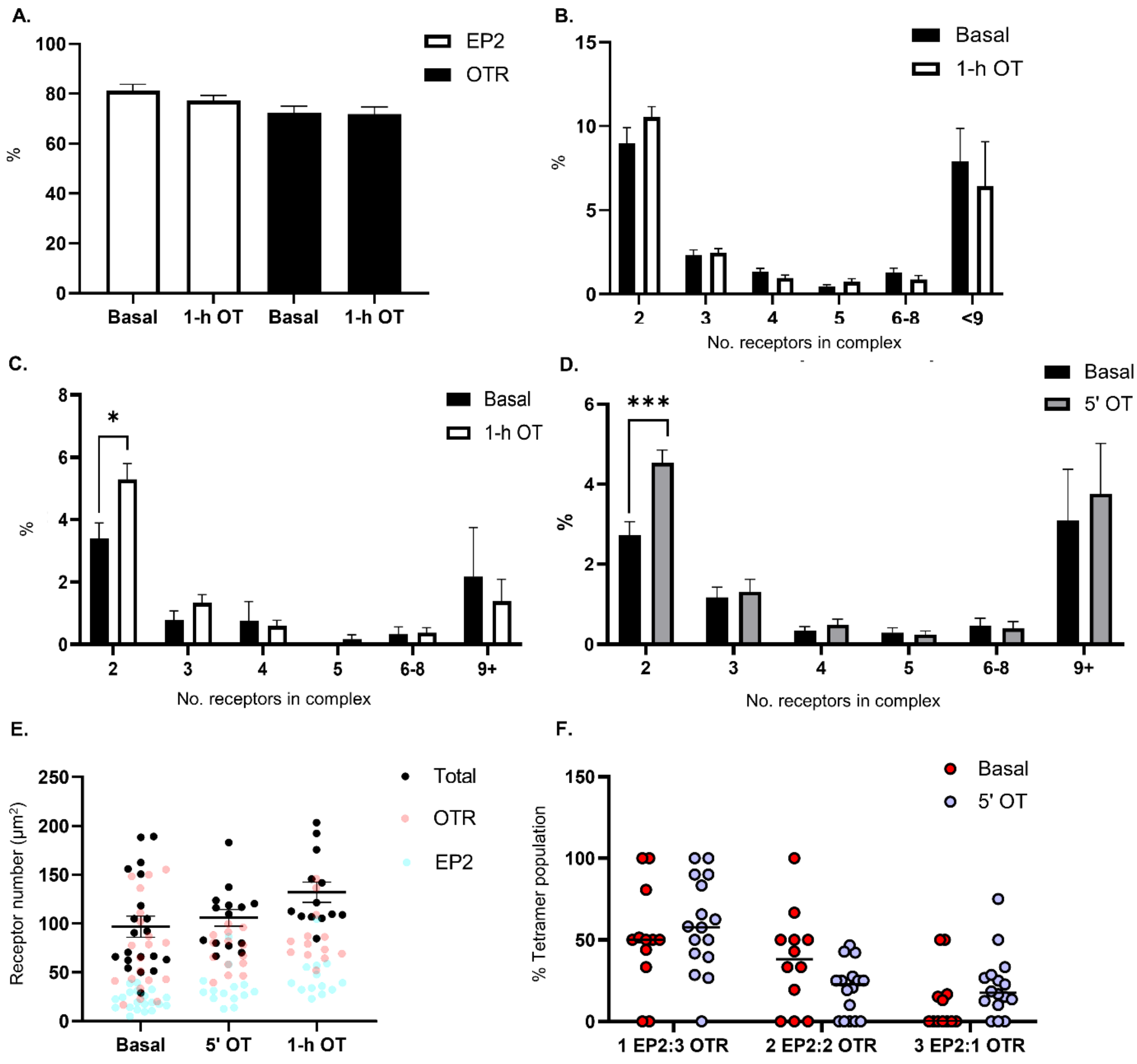

Figure S5. The EP2 and OTR cell surface monomer and homomer populations, related to Figure 5. PD-PALM analysis of cell surface EP2/OTR monomers and homomers in HEK 293 cells co-expressing FLAG-EP2 and HA-OTR with/without 1h OT stimulation (100 nM). EP2 and OTR monomer **(A)** and both OTR homomer (**B**), and EP2 homomer (**C)** populations were quantified from the same experiments presented in Fig. 5B as percentage of total EP2 or OTR, respectively, with/without 1h OT stimulation (100 nM). Data are mean ± SEM. n=4 independent experiments of 4-6 cells per experiment. Data represented as mean ± SEM. Unpaired *t* test: $p<0.05$.

(D) EP2-OTR heteromers with/without OT (100 nM, 5-minutes) expressed as % of all receptors, stimulated and imaged alongside cells presented in Fig. 5B. Mean ± SEM, n=4 independent experiments of 4-6 cells each. **(E)** Receptor number across PALM experiments from Fig. 5B and Supplementary Fig. S5D. **(E)** Composition of heterotetramers stimulated with/without OT (5-minute). Mean ± SEM, n=7, 2-6 cells each. *p< 0.05, ***P < 0.001, unpaired, two-tailed Student's *t*-test.

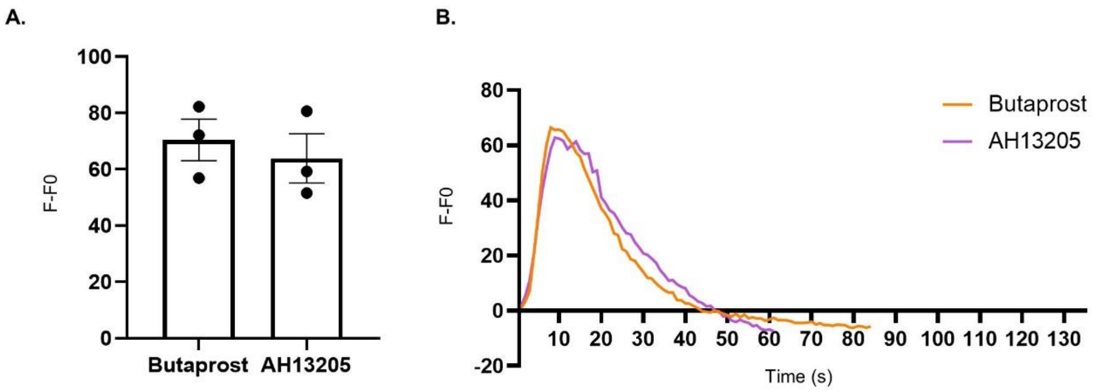

Figure S6. Butaprost and AH13205 induce Ca^{2+} release in non-laboring myocytes, related to Figure 6. Intracellular Ca^{2+} release in myocytes cultured from term pregnant non-laboring myometrium. Cells were imaged before and following stimulation with either AH13205 (10 µM) or butaprost (10 µM). **(A)** Data is the mean of the maximum fluorescent intensity of 30 cells per experiment normalized to unstimulated baseline (F- F0), shown as mean ± SEM. 30 cells were imaged in duplicate per sample, n=3.. **(B)** Representative fluorescent intensity traces over time from Fig. S6A.

Table S1. Accession numbers for RNAseq, Related to Figure 1.

Accession number	Labour type	Depository
ERR9539548	Early Induced	ENA
ERR9539547	Late	ENA
ERR9539546	Late Induced	ENA
ERR9539545	Late Induced	ENA
ERR9539544	Late Induced	ENA
ERR9539543	Late Induced	ENA
ERR9539542	Late Induced	ENA
ERR9539541	Late	ENA
ERR9539540	Late	ENA
ERR9539539	Late	ENA
ERR9539538	Late	ENA
ERR9539537	Early Induced	ENA
ERR9539536	Early Induced	ENA
ERR9539535	Early Induced	ENA
ERR9539534	Early Induced	ENA
ERR9539533	Early	ENA
ERR9539532	Early	ENA
ERR9539531	Early	ENA
ERR9539530	Early	ENA
ERR9539529	Early	ENA
ERR9539528	Non-laboring	ENA
ERR9539527	Non-laboring	ENA
ERR9539526	Non-laboring	ENA
ERR9539525	Non-laboring	ENA
ERR9539524	Non-laboring	ENA
