# Supplementary material for: Functional rewiring of G protein-coupled receptor signaling in human labor
Source: Cell Rep. 2022 Sep 6;40(10):111318. doi: 10.1016/j.celrep.2022.111318 (PMC9638024; doi:10.1016/j.celrep.2022.111318)
Supplement: Document S2. Article plus supplemental information [file mmc2.pdf]

Cell Reports

Article

Functional rewiring of G protein-coupled receptor signaling in human labor

Graphical abstract

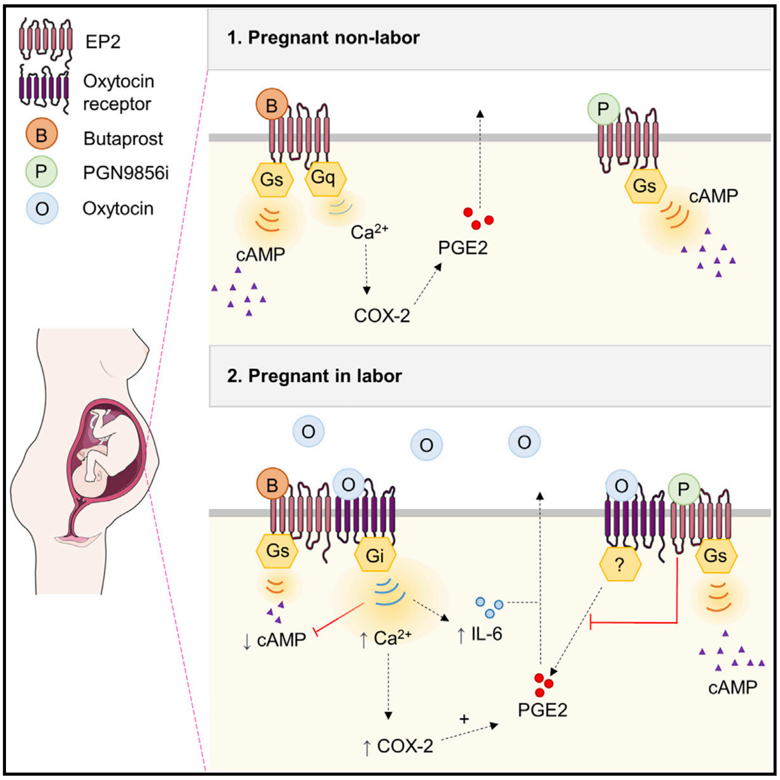

Authors

Abigail R. Walker, Camilla B. Larsen, Samit Kundu, ..., Shirin Khanjani, Phillip R. Bennett, Aylin C. Hanyaloglu

Correspondence

p.bennett@imperial.ac.uk (P.R.B.), a.hanyaloglu@imperial.ac.uk (A.C.H.)

In brief

The prostanoid receptor EP2 maintains myometrial quiescence during pregnancy. Walker et al. show that during human labor, EP2 signaling switches toward an inflammatory profile driven by functional crosstalk with the activated oxytocin receptor. An EP2 ligand, PGN9856i, is “resistant” to this crosstalk and could be exploited to delay preterm labor.

Highlights

- EP2 activity is reprogrammed toward pro-inflammatory pathways during human labor
- Oxytocin downregulates EP2-Gαs signaling and switches EP2-Gαq/11 signaling to Gαi/o
- EP2/OTR heterotetramers are reorganized by oxytocin to conformations favoring Gαi
- EP2 agonist PGN9856i does not activate pro-labor signals even after oxytocin treatment

Walker et al., 2022, Cell Reports *40*, 111318
September 6, 2022 © 2022 The Author(s).
https://doi.org/10.1016/j.celrep.2022.111318

Cell Reports

CellPress
OPEN ACCESS

Article

Functional rewiring of G protein-coupled receptor signaling in human labor

Abigail R. Walker,[1] Camilla B. Larsen,[1] Samit Kundu,[1,2] Christina Stavrinidis,[1] Sung Hye Kim,[1,2] Asuka Inoue,[3] David F. Woodward,[4] Yun S. Lee,[1,2] Roberta Migale,[1,5] David A. MacIntyre,[1,2] Vasso Terzidou,[1,2] Francesca Fanelli,[6,7] Shirin Khanjani,[1,8] Phillip R. Bennett,[1,2,*] and Aylin C. Hanyaloglu[1,9,*]

[1]Institute of Reproductive and Developmental Biology, Department Metabolism, Digestion and Reproduction, Imperial College London, London, UK
[2]March of Dimes European Preterm Birth Research Centre, Imperial College London, London, UK
[3]Graduate School of Pharmaceutical Sciences, Tohoku University, Sendai, Japan
[4]Department of Bioengineering, Imperial College London, London, UK
[5]Stem Cell Biology and Developmental Genetics Laboratory, The Francis Crick Institute, London, UK
[6]Department Life Sciences, University of Modena and Reggio Emilia, via Campi 103, 41125 Modena, Italy
[7]Center for Neuroscience and Neurotechnology, University of Modena and Reggio Emilia, via Campi 287, 41125 Modena, Italy
[8]Reproductive Medicine Unit, University College London Hospital, London, UK
[9]Lead contact
*Correspondence: p.bennett@imperial.ac.uk (P.R.B.), a.hanyaloglu@imperial.ac.uk (A.C.H.)
https://doi.org/10.1016/j.celrep.2022.111318

SUMMARY

Current strategies to manage preterm labor center around inhibition of uterine myometrial contractions, yet do not improve neonatal outcomes as they do not address activation of inflammation. Here, we identify that during human labor, activated oxytocin receptor (OTR) reprograms the prostaglandin E2 receptor, EP2, in the pregnant myometrium to suppress relaxatory/Gαs-cAMP signaling and promote pro-labor/inflammatory responses via altered coupling of EP2 from Gαq/11 to Gαi/o. The ability of EP2 to signal via Gαi/o is recapitulated with *in vitro* OT and only following OTR activation, suggesting direct EP2-OTR crosstalk. Super-resolution imaging with computational modeling reveals OT-dependent reorganization of EP2-OTR complexes to favor conformations for Gαi over Gαs activation. A selective EP2 ligand, PGN9856i, activates the relaxatory/Gαs-cAMP pathway but not the pro-labor/inflammatory responses in term-pregnant myometrium, even following OT. Our study reveals a mechanism, and provides a potential therapeutic solution, whereby EP2-OTR functional associations could be exploited to delay preterm labor.

INTRODUCTION

Worldwide, approximately 15 million babies/year are born preterm (<37 weeks of pregnancy), placing them at increased risk for adverse health and developmental difficulties (Chawanpaiboon et al., 2019). Preterm birth is the leading global cause of mortality in children under the age of 5, and although in most developed countries the rate of preterm birth is increasing, strategies to prevent or manage preterm labor are severely limited (Chawanpaiboon et al., 2019; Walani, 2020). Tocolytic drugs, intended to delay or inhibit preterm delivery, are widely used in the management of preterm labor but with limited benefits. Betamimetics, calcium channel blockers, prostaglandin inhibitors, and oxytocin receptor blockers have each been shown to have the potential to delay delivery but not to have any effect upon short- or long-term neonatal outcomes. Use of betamimetics has largely been abandoned because of serious maternal side effects, such as tachycardia, hyperglycemia, pulmonary edema, and cardiac arrhythmia (Lamont and Jørgensen, 2019; Lamont et al., 2016). Magnesium sulfate has seen a resurgence in use due to the documented neuroprotective effect for the preterm infant, however, there is no evidence that it has any useful tocolytic value (Crowther et al., 2014). Atosiban, a selective oxytocin (OT)/vasopressin receptor antagonist that is available for clinical use in Europe, but not in the USA, is a biased ligand with the potential to activate inflammation (Kim et al., 2016), which is not a desirable effect of tocolytics.

OT plays a central role during human parturition by driving contractions in the uterine smooth muscle or myometrium. OT exerts its actions via its receptor (OTR), a member of the superfamily of G protein-coupled receptors (GPCRs), representing the largest and most diverse group of signaling receptors and the current target of ~34% of prescribed drugs (Hauser et al., 2017). Activation of OTR in the myometrium results in activation of both Gαq/11 and Gαi/o heterotrimeric G protein pathways. OTR-mediated Gαq/11 signaling is predominantly responsible for eliciting forceful contractions in the pregnant myometrium, while Gαi/o signaling promotes inflammation (Kim et al., 2016).

© 2022 The Author(s).
This is an open access article under the CC BY license (http://creativecommons.org/licenses/by/4.0/).

Inflammatory pathway activation in the fetal membranes and myometrium leads to enhanced sensitivity to OT and increased synthesis of pro-inflammatory and pro-contractile prostaglandins, specifically PGE2 and PGF2α, which also activate distinct GPCRs. While GPCRs represent excellent candidate targets for drug development for the delay or prevention of preterm labor, their signaling pathways are complex and can be reprogrammed during human labor. For example, the PGE2 receptor EP2 is a well-described Gαs-coupled receptor that acts to increase cellular levels of the second messenger cAMP promoting uterine relaxation (Slater et al., 2006; Sugimoto and Narumiya, 2007). However, we have shown that in non-laboring term myometrium, EP2-selective agonists also activate a Gαq/11-calcium pathway to mediate pro-inflammatory cyclooxygenase-2 (COX-2) signaling, in addition to inhibiting myometrial contractility (Kandola et al., 2014). Following the onset of labor, the cAMP pathway is downregulated, while the pro-labor/inflammatory pathway is maintained (Kandola et al., 2014). Thus, a mechanistic understanding of the complex changes in GPCR signaling during human parturition will provide crucial insight for accelerating development of effective therapeutic strategies for preterm labor management.

In the present study, we identify both a translatable target in pregnant human myometrium and a compound with distinct properties to be exploited in preterm labor management. We show that activation of OTR reprograms G protein signaling of EP2 during human labor to drive pro-inflammatory responses. Through biophysical, super-resolution imaging and computational structural modeling, we demonstrate that heteromeric interactions of OTR and EP2 are modified following OTR activation to favor Gαi coupling. Finally, we show that a non-prostanoid EP2 ligand, PGN9856i, does not activate EP2-mediated pro-labor inflammatory signaling, a property that is not altered by OTR crosstalk and thus represents a promising candidate for preterm labor management.

RESULTS

Labor alters the signaling profile of EP2

We have previously demonstrated that EP2 signals via both Gαs/cAMP (pro-quiescence) and Gαq/11/COX-2 (pro-labor) pathways in term-pregnant human myometrium and that following labor onset, there is maintenance of pro-labor pathway activation but decreased cAMP signals (Kandola et al., 2014). To further understand how labor reprograms EP2 activity, we assessed these two functionally opposing pathways at distinct stages of labor. Myometrial samples were taken from women delivering by Caesarean section during early (cervical dilation <3 cm) or late labor (cervical dilation >3 cm) (Stanfield et al., 2019), with or without labor induction using intravenous OT (syntocinon). Primary myocyte cultures established from these samples were stimulated with either butaprost, a well-characterized EP2-selective agonist, or isoproterenol, a Gαs-coupled β-adrenergic receptor (βAR) agonist. Despite the previously reported downregulation of the Gαs pathway during labor (Europe-Finner et al., 1994), only butaprost-induced, but not isoproterenol-induced, cAMP signaling was progressively and significantly attenuated in all laboring groups (Figure 1A). In contrast, butaprost-induced increases in COX-2 were maintained across non-laboring and laboring groups, with only the early laboring group induced with syntocinon exhibiting a significant increase (Figures 1B and 1C).

To determine if these changes in EP2 signaling during labor were due to changes in gene expression of the receptor and/or its key heterotrimeric G protein pathways, RNA sequencing (RNA-seq) of the same patient samples was used to quantify the level of expression of EP2 (Ptger2) and distinct Gα subunits GNAS and GNAQ/GNA11. Analysis of OTR (Oxtr) transcript levels and one of its G protein pathways GNAI/O was also included, as the OTR signal system is a key driver of labor. There was no significant difference in the expression levels of EP2, GNAS, GNAQ/11, and OTR between early and late laboring samples, although there was a small increase in the expression of GNAI3 in laboring samples (Figure S1). This is consistent with our previous findings that EP2 mRNA is not changed in upper or lower segment myometrium during labor (Kandola et al., 2014). A correlation matrix between functional data presented in Figures 1A–1C and RNA levels revealed no significant correlation between fold change in cAMP and expression of GNAS or EP2/Ptger2, confirming that the reduction in butaprost-induced cAMP is not a product of altered EP2 or Gαs gene expression (Figure 1D). Similarly, no significant correlation was found between expression of the eight genes examined and fold change of butaprost-induced COX-2 levels (Figure 1D).

Together, these results indicate that EP2 activity is altered to favor a pro-inflammatory pathway over the anti-labor cAMP pathway at each stage of labor, representing a targeted reprogramming of EP2 over other Gαs-coupled GPCRs. A lack of transcriptional regulation of EP2, GNAS, or GNAQ/11 may indicate alterations in coupling of EP2 at the receptor/G protein level.

Incubation of non-laboring myometrium with OT reprograms EP2 signaling

As all patients in the laboring groups were exposed to OT *in vivo*, either through labor induction protocols or via release of endogenous hormones during spontaneous labor, we assessed whether *in vitro* OTR activation by OT is alone sufficient to alter the EP2 signal profile. We used an *in vitro* OT pretreatment time of 1 h to allow measurement of EP2-mediated acute upstream second messenger signal responses prior to significant induction of downstream inflammatory responses. Non-laboring, term myocyte cultures pretreated with or without OT were washed and stimulated with either butaprost or isoproterenol, and levels of intracellular cAMP were measured. While butaprost-induced cAMP signaling was significantly inhibited by OT pretreatment, isoproterenol-induced β2AR signaling was not affected (Figures 2A and 2B), consistent with cAMP responses observed in patient samples obtained following labor onset (Figure 1A). This selective inhibition, together with RNA-seq data, suggests that OT/OTR, which is both Gαq/11 and Gαi/o coupled, targets EP2 signaling potentially via OTR-Gαi/o-coupled pathways. To confirm this, myocytes were pretreated with the Gαi/o selective inhibitor pertussis toxin (PTX), which catalyzes the ADP ribosylation of Gαi/o subunits. We have previously demonstrated that in non-laboring myocytes, EP2 elicits

CellPress
OPEN ACCESS

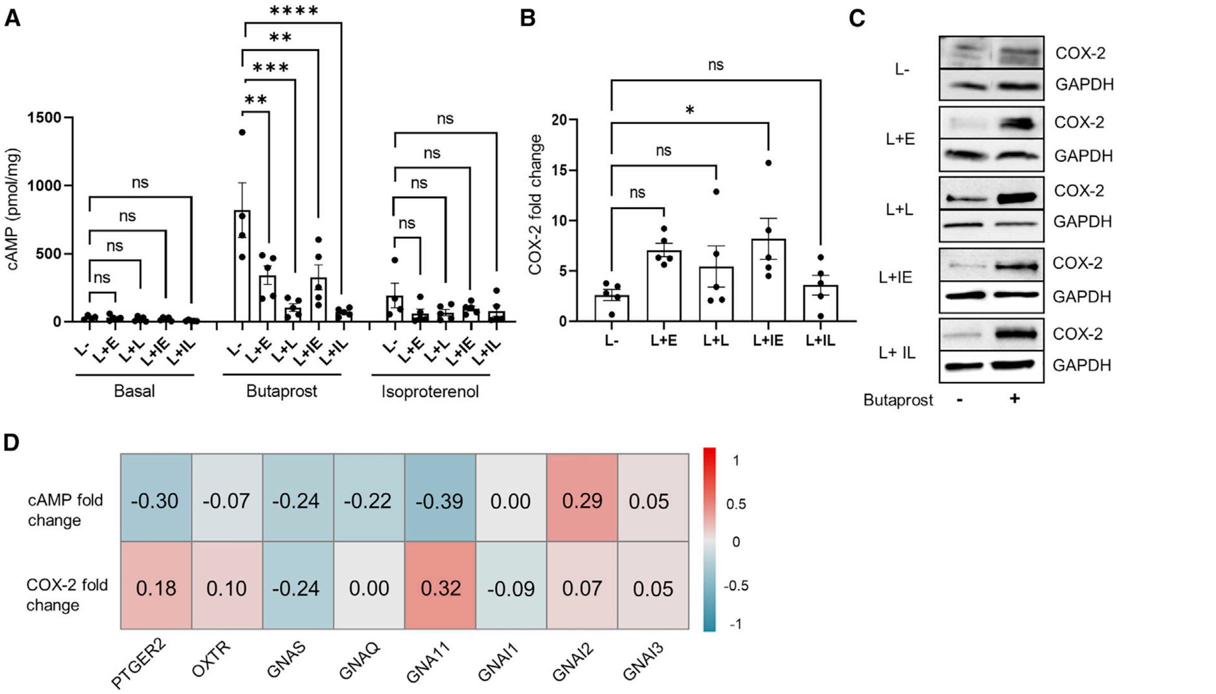

Figure 1. EP2 signaling in pregnant human myocytes is altered during labor to favor pro-inflammatory pathways

Myocytes cultured without passage (P0) were established from term-pregnant human myometrium from the following groups: L−, non-laboring; L + E, early-stage spontaneous labor; L+L, late-stage spontaneous labor; L + IE, early-stage induced labor; L + IL, late-stage induced labor.

(A) Intracellular cAMP measurements following stimulation with either butaprost (5 min, 10 μM) or isoproterenol (5 min, 10 μM). Data are shown as mean ± SEM; L− n = 4. All other groups, n = 5. One-way ANOVA with Dunnett's post-hoc test: $^{**}p < 0.01$, $^{***}p < 0.001$, $^{****}p < 0.0001$.

(B) COX-2 protein (70 kDa) levels determined by western blot and normalized to GAPDH (38 kDa) following treatment with butaprost (6 h, 10 μM). Data are shown are fold change over basal, mean ± SEM, n = 5. One-way ANOVA with Dunnett's post-hoc test: $^{*}p < 0.05$.

(C) Representative western blot corresponding to (B).

(D) Correlation matrix and correlation coefficients for functional data from all patients in (A)–(C) with RNA-seq-derived normalized expression levels of EP2 (Ptger2), OTR (Oxtr), and Gα subunits GNAS, GNAI/O, and GNAQ/11 quantified via RNA-seq.

See also Figure S1.

intracellular calcium (Ca^{2+} release via Gαq/11-dependent mechanisms; Kandola et al., 2014). In agreement with our previous findings, butaprost-induced Ca^{2+} release was not inhibited in PTX-treated conditions (Figures 2C and 2D). However, OT-pretreatment enhanced butaprost-mediated Ca^{2+} responses, and critically, this increase was reversed by PTX (Figures 2C and 2D). Functional crosstalk between these two receptors occurs in a unilateral manner, as pretreatment of myocytes with butaprost did not significantly affect the maximal response or temporal profile of OT-induced intracellular Ca^{2+} responses (Figure S2).

The EP2 agonist-mediated increase in COX-2 levels in term non-labor myocytes (without OT pretreatment) was also not significantly affected by PTX (Figures 2E and 2F). We have previously reported this pro-labor/inflammatory-associated pathway activated by EP2 is Gαq/11 mediated (Kandola et al., 2014). However, following OT pretreatment, butaprost-induced COX-2 levels were significantly enhanced, and furthermore, this increase was inhibited by PTX (Figures 2E and 2F). To determine if OT altered additional labor-associated inflammatory pathways downstream of EP2, secretion of the cytokine interleukin-6 (IL-6) was measured. Butaprost simulation alone increased IL-6 levels from 6 to 13 pg/mL, which was markedly upregulated to 160 pg/mL following OT pretreatment (Figure 2G). However, the butaprost-induced response was only sensitive to PTX following OT pretreatment (Figure 2G). Overall, these data indicate that OT activation of OTR modifies the G protein coupling of EP2 to Gαi/o, leading to promotion of inflammatory responses.

To confirm that this reprogramming of EP2 to Gαi/o occurs during labor *in vivo*, myometrial samples were taken following the onset of labor. Both upstream (intracellular Ca^{2+}) and downstream (COX-2 and IL-6) pro-labor pathways activated by butaprost were measured in the absence or presence of PTX (Figures 3A–3E). The ability of butaprost to induce increases in the pro-labor mediators measured (Ca^{2+}, IL-6) was significantly inhibited in PTX-treated conditions (Figures 3A–3C), which was not observed in non-laboring samples (Figure 2). Inter-patient variability in butaprost-induced levels of COX-2 in laboring samples was observed, although levels were significantly inhibited by PTX treatment (Figures 3D and 3E). These results confirm the acquired ability of EP2 to signal via Gαi/o to activate pro-inflammatory pathways in samples exposed to endogenous OT during labor, as well as following *in vitro* stimulation of non-laboring myocytes.

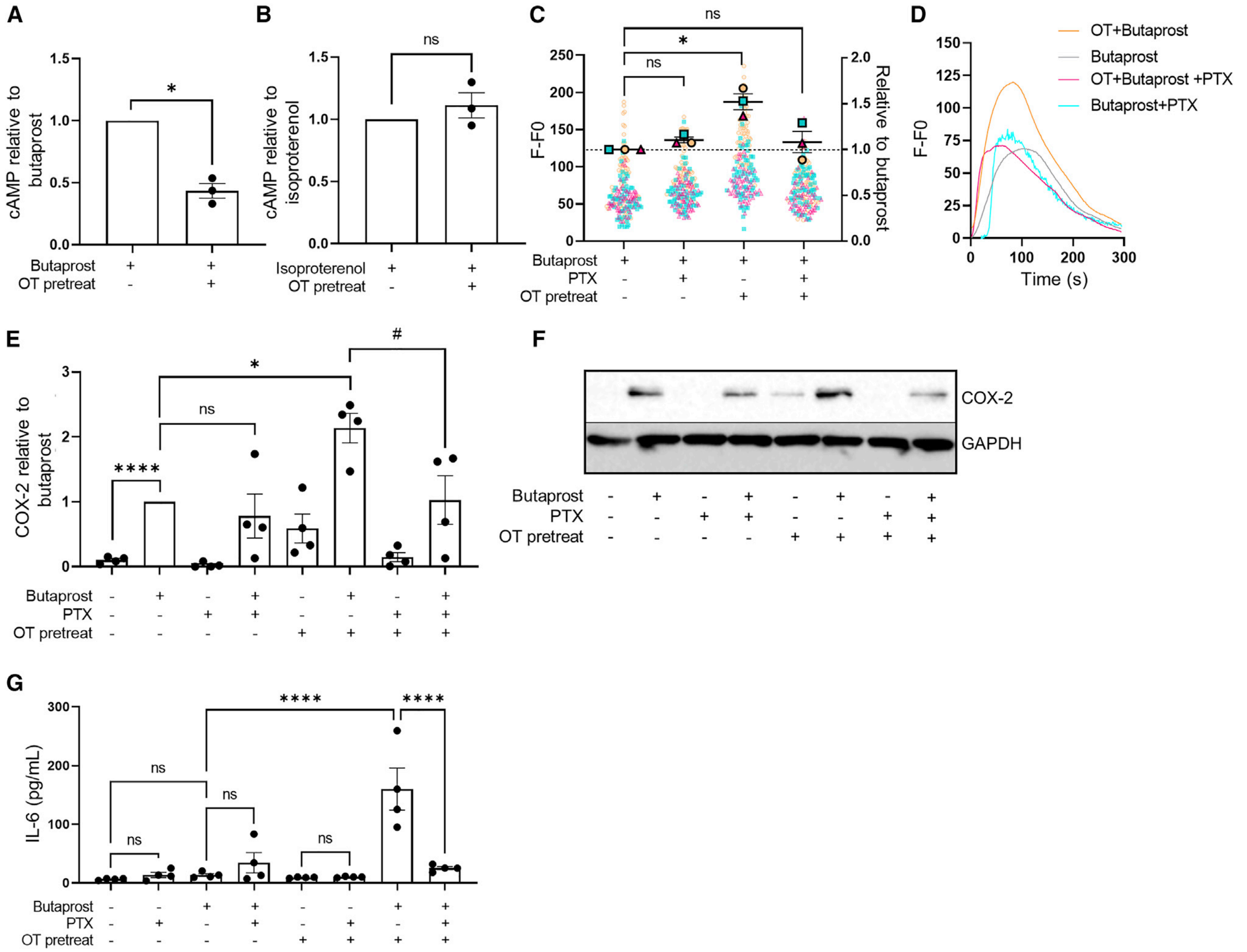

Figure 2. Oxytocin (OT) priming of non-laboring myocytes reprograms EP2-mediated pro- and anti-labor pathways via a Gαi/o mechanism

(A and B) Butaprost (10 μM) (A) and isoproterenol-mediated (10 μM) (B) cAMP response before and after 1 h OT pretreatment (100 nM). Data shown are normalized to butaprost or isoproterenol treatment, mean ± SEM, n = 3. *$p < 0.05$ by one-sample t test.

(C) Intracellular Ca^{2+} release following butaprost (10 μM) stimulation with/without pertussis toxin (PTX; 200 ng/mL) and/or 1 h OT (100 nM) pretreatment. Data are the maximal fluorescent intensity normalized to unstimulated baseline (F-F0) shown for each cell analyzed, overlaid with the mean of the maximum cell intensity per experiment ± SEM. Each cell analyzed is represented and color coded for each biological repeat. Data are shown relative to butaprost response ± SEM. n = 3.

(D) Representative fluorescent intensity traces from (C).

(E) Butaprost (10 μM, 6 h)-mediated upregulation of COX-2 protein (70 kDa) following with/without PTX and 1 h OT pretreatment, determined by western blot and normalized to GAPDH levels (38 kDa). Data are shown as fold change over basal, mean ± SEM, n = 4. One-sample t test: *$p < 0.05$, ****$p < 0.0001$. Unpaired t test: #$p < 0.05$.

(F) Representative western blot for (E).

(G) Release of IL-6 following 6 h butaprost (10 μM) stimulation with/without PTX and/or OT pretreatment. Data are shown as mean ± SEM, n = 4. One-way ANOVA with Sidak's post-hoc test: ****$p < 0.0001$.

See also Figure S2.

EP2-dependent activation of Gαi/o signaling requires activated OTR

Our data suggest that EP2 signaling is "rewired" from Gαq/11 to Gαi/o signaling during labor via crosstalk with the OTR. To directly determine if EP2 activation of Gαi/o signaling can only occur via an OTR-dependent mechanism, we used previously reported HEK 293 cells lacking endogenous Gαq/11 proteins (ΔGαq/11) (Grundmann et al., 2018). Butaprost-dependent Ca^{2+} signaling was measured in wild-type (WT) or ΔGαq/11 cells expressing either EP2 (Figures 4A and 4B) or both EP2 and OTR (Figures 4C and 4D). Flow cytometry confirmed the expression of FLAG-tagged EP2 at the surface to be at equivalent levels in both WT and ΔGαq/11 cells (Figure S3). Increases in intracellular Ca^{2+} were measured as we observed a switch to Gαi/o-dependent pathways to mediate EP2-dependent Ca^{2+} signaling in myocytes following the onset of labor (Figure 3A). Butaprost did not elicit a Ca^{2+} response in ΔGαq/11 cells expressing EP2 only, compared with WT HEK 293 cells, confirming its Gαq/11

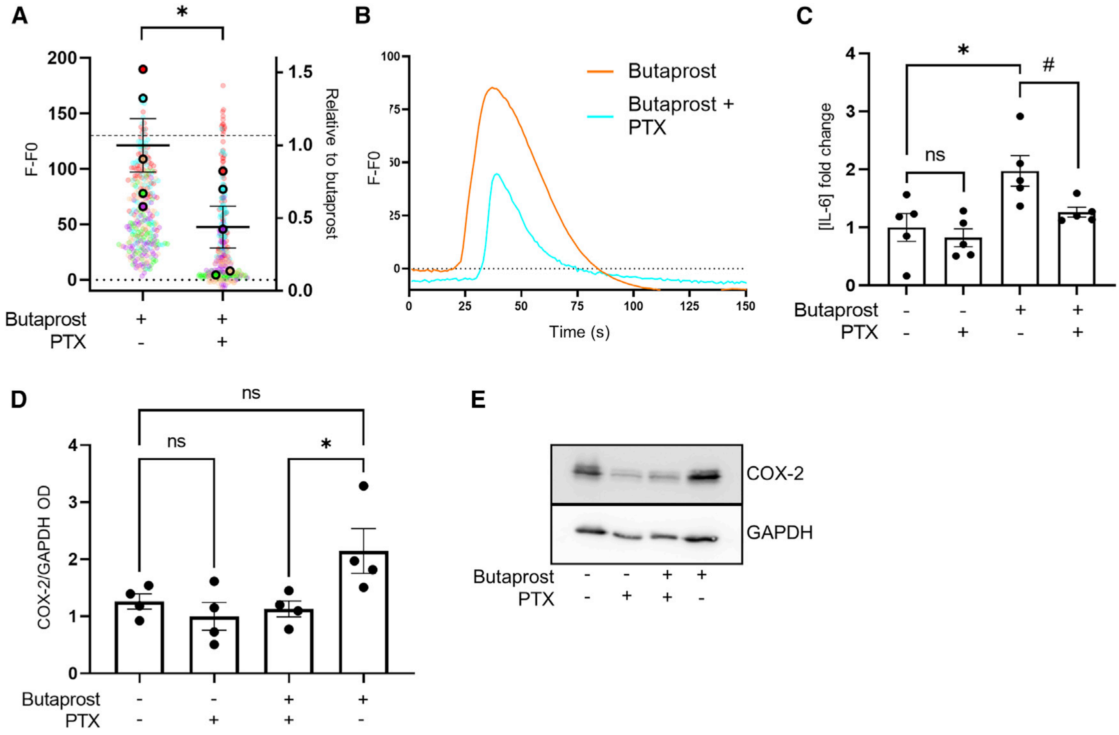

Figure 3. EP2 activates pro-labor pathways in laboring myocytes via a Gαi/o pathway

Myocyte cultures (P0) were obtained from term-pregnant, laboring myometrium.

(A) Myocytes were loaded with Fluo4-AM and imaged live via confocal microscopy. Butaprost (10 μM)-mediated intracellular Ca^{2+} release with/without PTX (200 ng/mL) pretreatment. Data are the maximal fluorescent intensity normalized to unstimulated baseline (F-F0) shown for each cell analyzed, overlaid with the mean of the maximum cell intensity per experiment shown relative to the butaprost-only response ± SEM. Variation within the butaprost-only response is also shown. Each cell analyzed is represented and color coded for each biological repeat, n = 5. One sample t test: $*p < 0.05$.

(B) Representative fluorescent intensity traces over time from (A).

(C) Release of IL-6 in laboring myometrial cells following 6 h butaprost (10 μM) stimulation with/without PTX (200 ng/mL). Data are shown as fold change over basal, including the variation within the basal, mean ± SEM, n = 5. One-sample t test: $*p < 0.05$. Unpaired t test: $\#p < 0.05$.

(D) Butaprost (10 μM)-mediated upregulation of COX-2 protein (70 kDa) with/without PTX pretreatment (200 ng/mL) determined by western blot and normalized to levels of GAPDH (38 kDa). Mean ± SEM, n = 4. One way ANOVA with Sidak's post-hoc test: $*p < 0.05$.

(E) Representative western blots for (D).

dependence for this pathway (Figures 4A and 4B). In ΔGαq/11 cells expressing both EP2 and OTR, butaprost stimulation did not elicit a Ca^{2+} signal until cells were pretreated with OT (Figures 4A and 4B). Critically, this rescue in EP2 agonist-dependent Ca^{2+} signaling was significantly inhibited by PTX pretreatment (Figures 4C and 4D). This suggests that the Gαi/o-dependent signaling of EP2 only occurs via OT-activated OTR/Gαi/o.

Super-resolution imaging of EP2/OTR heteromers reveal selective molecular complexes are modulated by OT

A potential mechanism for the crosstalk and reprogramming of EP2 signaling to a Gαi/o pathway is via formation of EP2/OTR heteromeric complexes. The ability of GPCRs to form heteromers with distinct GPCRs is a known mechanism for diversifying GPCR signaling *in vivo* to alter GPCR signaling and activity (Ferrada et al., 2009; Rozenfeld et al., 2011). Heteromers of EP2 and OTR were detected by bioluminescence resonance energy transfer (BRET), a biophysical approach to detect protein-protein interactions in living cells (Ayoub and Pfleger, 2010), in HEK 293 cells transiently expressing Rluc8-tagged EP2 and increasing levels of Venus-tagged OTR (Figure S4A). These experiments were also performed in primary human myocytes from term-pregnant myometrium (Figure S4B), and interaction of endogenous EP2 and OTR was detected in non-laboring myometrium both before and after OT treatment via proximity ligation assay (Figure S4C).

To provide detailed information on all possible EP2/OTR heteromers at the plasma membrane (including all receptor forms such as monomers), quantitation of EP2-OTR complexes was measured by dual-color photoactivated localization microscopy with photoactivatable dyes (PD-PALM). We have previously demonstrated that this super-resolution, single-molecule imaging approach can be used to quantitate the complex oligomerization properties of distinct GPCR homomers and heteromers (Buenaventura et al., 2019; Casarini et al., 2020; Jonas et al., 2015, 2018). PD-PALM provides a resolution of <10 nm, enabling visualization and quantitation of monomers, homo/heterodimers, and low-order homo/heterooligomers (homo/heterotrimers and homo/heterotetramers) and their distinct protomer compositions. As OT changes the EP2 signaling profile, we

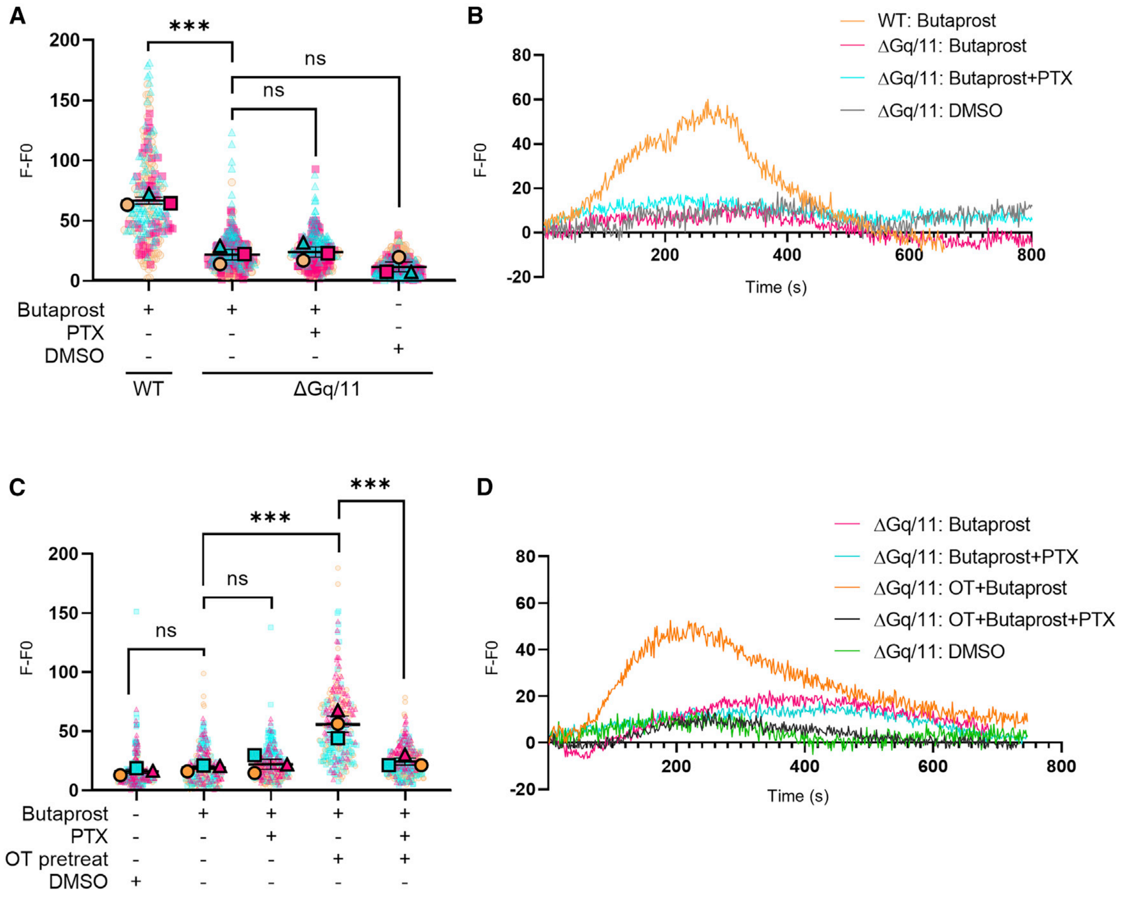

Figure 4. EP2-dependent Gαi/o signaling requires ligand-activated OTR

Intracellular Ca^{2+} release was measured in wild-type (WT) HEK 293 cells or HEK 293 cells lacking endogenous Gαq/11 proteins (ΔGαq/11). Cells were stimulated with vehicle (DMSO) or butaprost (10 μM) with/without 1 h OT pretreatment and/or PTX pretreatment (200 ng/mL).

(A) Cells expressing EP2. Data are the maximal fluorescent intensity normalized to unstimulated baseline (F-F0) shown for each cell analyzed, overlaid with the mean of the maximum cell intensity per experiment ± SEM. Each cell analyzed is represented and color coded for each biological repeat. n = 3 independent experiments. ANOVA with Sidak's post-hoc test: ***$p < 0.001$.

(B) Ca^{2+} intensity traces from representative cells from (A).

(C) Cells co-expressing EP2 and OTR. Maximal fluorescent intensity normalized to unstimulated cells as in (A).

(D) Representative Ca^{2+} intensity from (C). n = 3 independent experiments. ANOVA with Sidak's post-hoc test: ***$p < 0.001$.

See also Figure S3.

determined whether OTR activation induces rearrangement of specific EP2/OTR heteromeric complexes. PD-PALM imaging of receptors at the plasma membrane was carried out in HEK 293 cells expressing FLAG-tagged EP2 and hemagglutinin (HA)-tagged OTR, using anti-FLAG and -HA antibodies directly conjugated with CAGE dyes 500 and 552 as we have previously characterized (Jonas et al., 2015) (Figure 5A). PD-PALM analysis revealed that ~13% of EP2 and OTR molecules at the plasma membrane were in preformed EP2-OTR heteromeric complexes, with the remaining comprising of homomers or monomers (Figures 5B and S5A–S5C). The monomeric population of EP2 and OTR (Figure S5A) was higher than previously observed for GPCRs such as luteinizing hormone receptor (LHR) and follicle-stimulating hormone receptor (FSHR), and more consistent with single-molecule imaging studies of other class A GPCRs (Calebiro et al., 2013; Kasai et al., 2011). Within the heteromeric population, the primary preformed OTR/EP2 complex was heterodimers (~55% of the heteromers); however, a range of low- to higher-order heterooligomers was also observed (Figure 5C). There was a significant increase within the heterodimer population following pretreatment with OT following either 1 h, as employed for functional studies (Figure 5B), or a more acute time point of 5 min (Figure S5D) with no change in the overall surface levels of either OTR or EP2 at the plasma membrane (Figure S5E). We have previously demonstrated that GPCR complexes with >6 receptors are density dependent, while low-order GPCR di/oligomers (2–5 receptors) are not (Jonas et al., 2015); thus, low-order heterooligomers were further analyzed for protomer composition. Quantification of the stoichiometry of EP2 and OTR protomers within the heterotrimers and heterotetramers following either 5 min or 1 h OT treatment indicated that only the heterotetramer composition

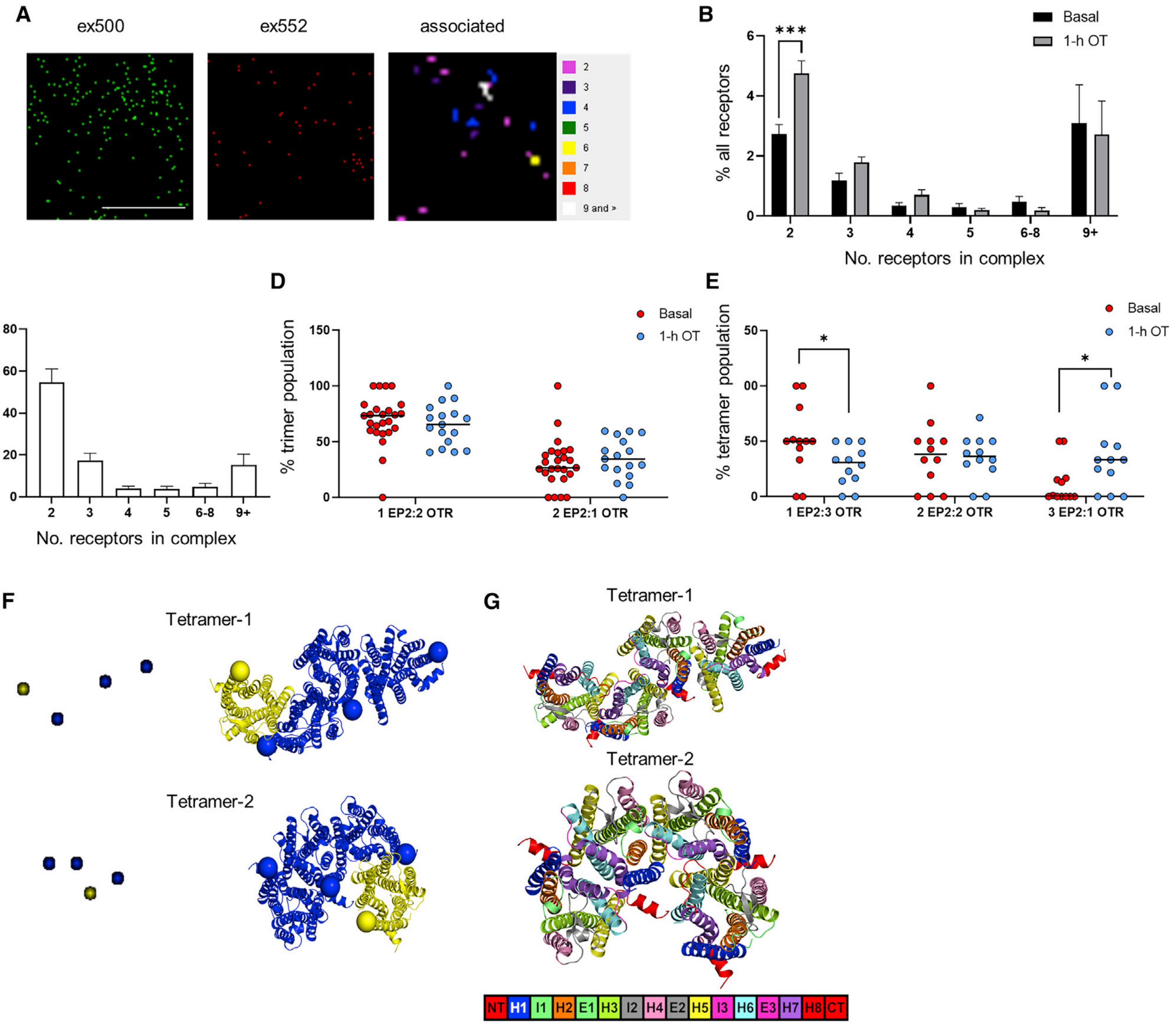

Figure 5. EP2-OTR heterotetramer complexes are reorganized following OTR activation

(A) Representative PD-PALM super-resolution images of FLAG-EP2 labeled with CAGE 500 (ex500) and HA-OTR labeled with CAGE 552 (ex552). Images are 2 μm^2 box; scale bar, 1 μM with heatmap of the number of associated molecules.

(B) EP2-OTR heteromers with/without OT (100 nM, 1h) expressed as percentage of all receptors. Mean ± SEM, n = 4 independent experiments of 4–6 cells each.

(C) Preformed EP2-OTR complexes as percentage of total heteromers from (B). Mean ± SEM.

(D and E) Composition of heterotrimers (D) and heterotetramers (E) stimulated with/without OT. Mean ± SEM, basal n = 7, 2–6 cells each, OT treated n = 4, 3–4 cells each. *$p < 0.05$, ***$p < 0.001$, unpaired, two-tailed Student's t test.

(F) PD-PALM images (left) concerning the most recurrent spatial arrangements of 2 activated OTR (yellow) and EP2 (blue) heterotetramers are shown adjacent to the structural models whose architectures closely align with PD-PALM images. Yellow (OTR) and blue (EP2) spheres are centered on the Cα-carbon atom of the first amino acid.

(G) The same structural models as in F are colored according to helices (H), extracellular loops (E), and intracellular loops (I) (see legend bar). Numbers highlight the helices at the interface between OTR and EP2.

See also Figures S4 and S5.

was altered following 1 h OT pretreatment (Figures 5D, 5E, and S5F), suggesting that these higher-order heteromers may be more dynamic. Basally, ~60% of tetramers consisted of the 1EP2:3OTR complex; however, following OT treatment, there was a rearrangement in protomer composition within these complexes to change the asymmetry toward EP2, as evidenced by the significant increase in 3EP2:1OTR and significant decrease in the levels of 1EP2:3OTR tetramers (Figure 5E). Overall, these data indicate that EP2 and OTR form diverse heteromeric assemblies and that OT stimulation changes both the level of these heteromers and reorganization of the protomers within a complex.

Predictions of EP2/OTR heteromeric complexes via molecular modeling, according to an established protocol (Casciari et al., 2006; Fanelli et al., 2013; Jonas et al., 2015), show substantial alignment with PD-PALM images. This further supports that organization of receptors detected via PD-PALM is due to contacts between selected transmembrane (TM) helices. Alignment of PD-PALM imaged complexes with modeling suggested that the most recurrent interfaces include the following TM helices (Hs): (1) H4-H7,H1; H5-H6; (2) H4-H5; H5-H4; (3) H1-H7; H4-H5; (4) H4-H1; H1-H4; (5) H5-H7; H7-H5; H6-H6; (6) H4-H4; and (7) H1-H1. Interestingly, the active state of OTR uses H6 less frequently at the interface than the inactive state because of the peculiar outward tilt of the helix upon activation. In general, the active OTR protomer (OTR*) limits the number of possible heteromer architectures compared with inactive OTR. The most recurrent 3EP2:1OTR* architectures are depicted in Figure 5F. For two (in tetramer-1) or all (tetramer-2) EP2 protomers, H6 cannot undergo outward movements for G protein coupling, as it is involved in the protomer interface (Figure 5F). It has been hypothesized that less-pronounced outward movements of H6 in Gαi-coupled rod opsin, compared with the Gαs-coupled β2AR, might determine receptor-G protein coupling specificity (Kang et al., 2018). The architecture of tetramer-2 is such that only OTR* can couple with G protein and that, similarly to tetramer-1, the allowable outward tilt of H6 in OTR* is more suitable to Gαi coupling. Collectively, the combination of PD-PALM imaging and structural modeling suggests that pretreatment with OT favors the formation of 3EP2:1OTR* tetramers whose preferred architecture tends to bias toward OTR-mediated Gαi signaling.

Bypassing OTR/EP2 crosstalk via a non-prostanoid EP2 agonist

Our findings suggest that during labor, OTR reprograms EP2's G protein signaling to promote pro-inflammatory responses. Current tocolytic drugs that target OT/OTR signaling, however, aim to temporarily inhibit uterine contractions, primarily via antagonizing OTR without considering the pro-inflammatory actions of OTR nor the amplification of these pathways via crosstalk with distinct GPCRs. Thus, EP2 ligands that could be resistant to this reprogramming by OTR during labor may offer a therapeutic strategy in preterm labor management. PGN9856i is a recently reported, highly selective EP2 agonist that is more potent at inhibiting human myometrial contractions than butaprost and produces exceptionally long-lasting reductions in intraocular pressure (Bertrand et al., 2020; Coleman et al., 2019). PGN9856i treatment activated Gαs/cAMP signaling in EP2-expressing HEK 293 cells with a half maximal effective concentration (EC50) of 2.11 nM (95% confidence interval [CI] 0.11–8.08 nM) (Figure 6A) but, unlike butaprost, did not increase intracellular Ca^{2+} (Figure 6B). In non-laboring, term-pregnant myometrial cells, PGN9856i activated cAMP to similar levels as butaprost (Figure 6C). However, PGN9856i did not increase intracellular Ca^{2+}, including after OT pretreatment, which significantly enhanced butaprost-induced signaling (Figures 6D and 6E). The ability of EP2 agonists to induce Ca^{2+} signaling is not a property of butaprost alone, as the less-potent but selective EP2 ligand AH-13205 (Nials et al., 1993) can also induce Ca^{2+} signaling in myocytes (Figure S6), confirming the distinct properties of PGN9856i among the EP2 agonists tested. We have previously demonstrated that EP2-mediated Ca^{2+} signaling drives COX-2 and PGE2 release, an inflammatory mediator downstream of COX-2 activation (Kandola et al., 2014). While butaprost-mediated PGE2 release in myometrial cells was significantly increased by OT, PGN9856i was unable to stimulate PGE2 release with or without OT pretreatment (Figure 6F). Interestingly, in those patient samples that exhibited increases in PGE2 release following OT pretreatment, PGN9856i significantly reduced this increase in PGE2 (Figure 6G). Together, these results identify an EP2 ligand with a signal profile in pregnant human myocytes that potentially modulates labor/OT-driven inflammatory responses.

DISCUSSION

Labor is commonly described as an inflammatory cascade, yet this pathway is not targeted by most tocolytic drugs employed. The importance of inflammatory signals in the pregnant human myometrium is highlighted by our current findings demonstrating that during labor, OTR can program selective receptor systems toward pro-inflammatory pathways, representing an unexplored therapeutic opportunity. By identifying the mechanistic pathway of receptor crosstalk between OTR and EP2, we in turn identify therapeutic targeting strategies to prevent or delay preterm labor. Critically, we provide evidence for a therapeutic solution where an EP2-selective agonist can selectively activate relaxatory signals while also resisting functional crosstalk toward pro-inflammatory pathways by active OTR.

Functional and RNA-seq analysis in term, pregnant human myometrial samples, before and during different stages of labor, demonstrated that the EP2 signaling profile is specifically targeted at an upstream level, over other Gαs-coupled receptors, to promote pro-inflammatory signals over relaxatory, pro-quiescence cAMP signals. OT *in vitro* stimulation was sufficient to induce this targeted reprogramming of EP2, with the resulting crosstalk between EP2 and OTR "rewiring" the heterotrimeric G protein of EP2 from Gαq/11 signaling to Gαi/o signaling. This altered coupling enhanced EP2-mediated pro-inflammatory pathways. The ability of OT to inhibit butaprost, but not isoproterenol, responses further support that it is not only due to activation of the Gαi/o-coupled OTR and more global cellular inhibition of cAMP signaling from Gαs-coupled receptors in the myocyte, but a targeted regulation of EP2 signaling toward Gαi/o signaling. We have recently demonstrated that OTR and the PGF2α receptor may cooperate in promoting contractility induced by either OT or PGF2α (Kim et al., 2019) and now demonstrate an additional mechanism of labor promotion whereby OTR engages mechanisms to rewire the upstream signaling of a distinct GPCR, classically associated as a relaxatory/anti-labor receptor, to promote pro-inflammatory responses via Gαi/o. Such labor-driven alterations in the myometrium may also be facilitated at the transcriptional level, as RNA-seq analysis on the same patient samples used for functional studies had increased expression of GNAI3 during labor, further supporting a role for the Gαi/o pathway. An additional feature of this OTR/EP2 crosstalk is in the functional asymmetry,

CellPress
OPEN ACCESS

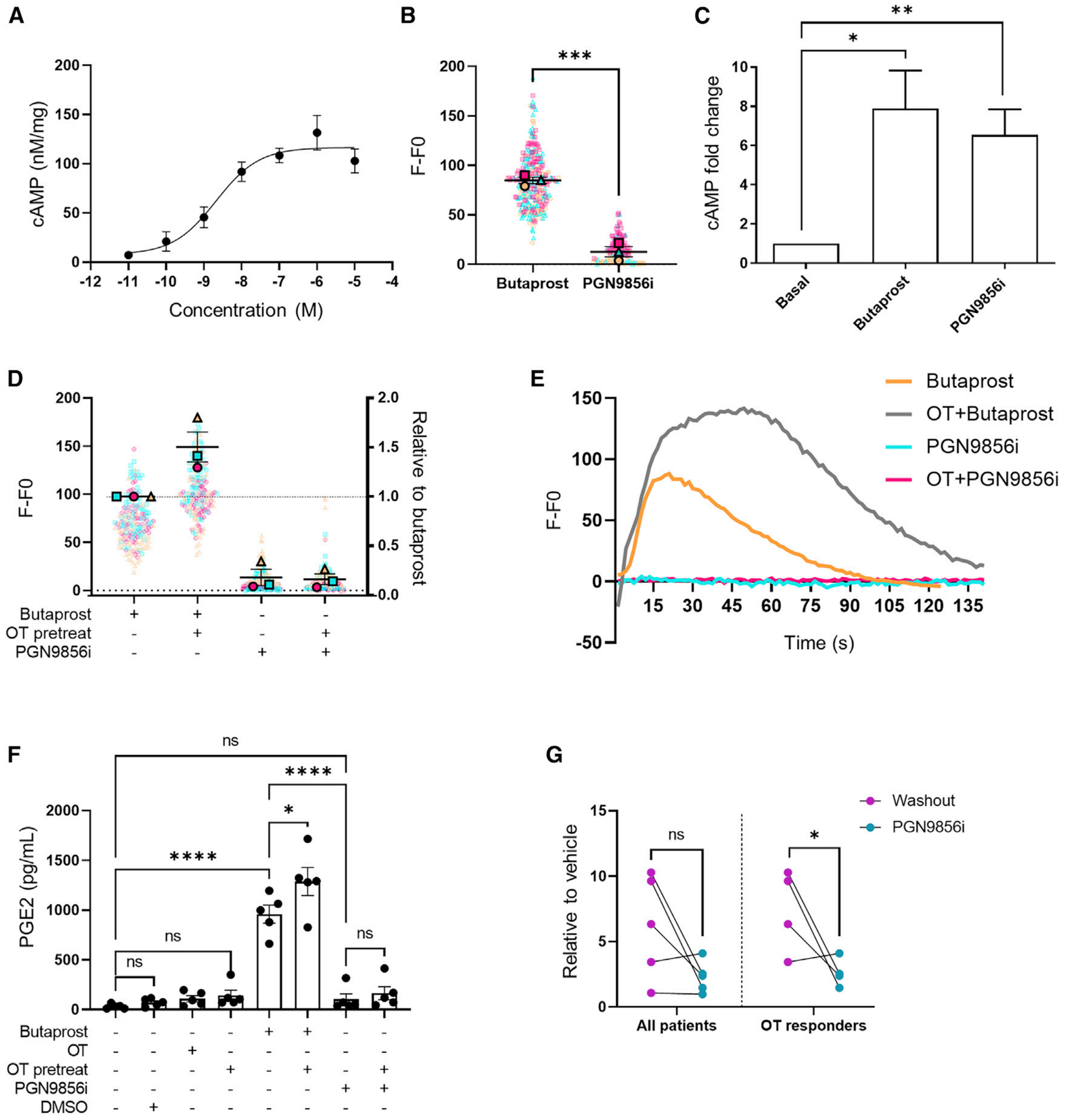

Figure 6. PGN9856i does not activate pro-labor pathways of EP2 and is resistant to modulation by activated OTR

(A) HEK 293 cells stably expressing FLAG-EP2 were stimulated with PGN9856i (0.01 nM–10 μM, 5 min), and cAMP levels were measured and normalized to protein. Data represent mean ± SEM, n = 3.

(B) Intracellular Ca^{2+} release in HEK 293 cells stably expressing human EP2. Cells were imaged before and following stimulation with either PGN9856i (100 nM) or butaprost (10 μM). Data are the maximal fluorescent intensity normalized to unstimulated baseline (F-F0) shown for each cell analyzed, overlaid with the mean of the maximum cell intensity per experiment ± SEM. Each cell analyzed is represented and color coded for each biological repeat, shown as mean ± SEM. Thirty cells were imaged in duplicate per sample, n = 3. p value calculated by unpaired Student's t test, ***$p < 0.001$.

(C) cAMP levels in non-laboring primary myometrial cells stimulated with either butaprost (10 μM) or PGN9856i (100 nM). Data are shown as fold change over basal, mean ± SEM, n = 7. *$p < 0.05$, one-sample t test.

(D) Intracellular Ca^{2+} levels in non-laboring myometrial cells following butaprost or PGN9856i with/without OT pretreatment (1 h, 100 nM). Data are the maximal fluorescent intensity normalized to unstimulated baseline (F-F0) shown for each cell analyzed, overlaid with the mean of the maximum cell intensity per experiment shown relative to the butaprost-only response ±SEM. Each cell analyzed is represented and color coded for each biological repeat. Mean ± SEM of fold change over butaprost, n = 3. One sample t test: **$p < 0.01$. Unpaired t test: ns, $p > 0.05$.

(E) Representative Ca^{2+} responses from (D).

(F) Secreted PGE2 in pregnant non-laboring myometrial cells following 6 h stimulation with either butaprost, PGN9856i, OT, or DMSO with/without OT pre-treatment. Mean ± SEM, n = 5. One way ANOVA with Sidak post-hoc test: *$p < 0.05$, ****$p < 0.0001$.

(G) PGE2 release from data in (F) normalized to vehicle. Left panel represents all patient data (n = 5), and right panel shows data only from patients who responded to OT in terms of increasing PGE2 (n = 4). Data are shown as mean ± SEM. Unpaired t test: *$p < 0.05$.

See also Figure S6.

whereby OT pretreatment can alter EP2 signaling, yet the EP2 agonist butaprost cannot enhance OTR responsiveness. In combination with our findings demonstrating rescue of EP2-mediated calcium signaling in the ΔGαq/11 cells, this suggests that receptor-receptor communication enables EP2 signaling to Gαi/o via an OT-activated OTR.

GPCR crosstalk often occurs via receptor heteromeric associations (Balenga et al., 2014; Callén et al., 2012), and OTR is known to associate with itself and other GPCRs (Albizu et al., 2010; de la Mora et al., 2016; Romero-Fernandez et al., 2013; Terrillon et al., 2003). This in turn raises the question of whether additional receptors are modulated by OTR during labor to promote both contractility and inflammation. This could be modulation of distinct GPCRs, other membrane receptors, or even crosstalk with nuclear receptors. Interestingly, the functional progesterone withdrawal that occurs prior to labor has been demonstrated to occur via crosstalk of membrane progesterone receptors to Gαi with nuclear progesterone receptors (Karteris et al., 2006). Thus, a potential upstream mechanism in the ability of OTR to selectively modulate distinct receptors may be facilitated by the altered activity of progesterone receptors or may, with the current study, indicate a more complex, dynamic network of receptor-receptor associations prior to and during labor.

In this study, we provide quantitation of the associations and organization between these receptors via super-resolution, single-molecule imaging (PD-PALM). While EP2 and OTR could form a range of distinct complexes, specifically, heterodimers were increased following OTR activation, and heterotetramers were reorganized upon OT activation to favor an asymmetric 3EP2:1OTR* complex. This reorganization of heterotetramers to favor asymmetric complexes with 3OTR:1EP2 specifically was evident under OT treatment conditions that alter EP2 signaling, yet following more acute OTR activation, all possible heterotetrameric combinations were present. This could indicate that these oligomeric complexes are highly dynamic and/or that formation of these specific higher-order complexes require additional processes such as reorganization into discrete microdomains that could further promote EP2 activation of Gαi signaling via OT/OTR. Interestingly, the recently reported OTR cryoelectron microscopy (cryo-EM) structure (Meyerowitz et al., 2022) has identified several cholesterol-binding sites in the receptor that could act as allosteric modulators in OTR signal complexes and/or indicate potential for plasma membrane microdomain organization. Overall, receptor-receptor acute allosteric modulations could integrate to induce these changes during the acute and chronic stages of labor. Alignment of PD-PALM data of the heterotetramers with computational modeling also supports a mechanism for receptor-receptor associations mediating the altered EP2 G protein signaling. It also provides a future model to understand how distinct ligands stabilize specific heteromeric complexes, resulting in different activity profiles of distinct EP2-selective ligands, butaprost and PGN9856i, following OTR activation.

PGN9856i was selected for study because it has properties not shared by other EP2 agonist molecules. PGN9856i is electrochemically neutral and directly targets EP2 to modulate established physiological functions of EP2, including inhibition of human myometrial contractions and, in ocular ciliary smooth muscle cells, reducing intraocular pressure (Bertrand et al., 2020; Coleman et al., 2019; Woodward et al., 2019). Interestingly, its ability to lower intraocular pressure lasts over several days from a single application (Bertrand et al., 2020; Woodward et al., 2019). In the current study, the distinct properties of PGN9856i, compared with other EP2 agonists tested, further extend to the functional profile induced in human term-pregnant myocytes. While current tocolytic strategies are only effective in delaying labor short term, if PGN9856i is found to inhibit other OTR-mediated pathways, targeting both inflammatory and contractile machinery, PGN9856i may be successful not only as an acute intervention but also as maintenance therapy for preterm labor. Neonatal and maternal outcomes might be further improved by combining PGN9856i with existing tocolytic and anti-inflammatory drugs.

In summary, our findings strongly support a model whereby distinct GPCR signal systems exhibit crosstalk, enabling OT and its receptor to orchestrate key pro-labor responses from other receptors. This evolved model of GPCR signaling highlights the need to reevaluate the strategy of targeting a single receptor system in the pregnant human myometrium but also that exploiting these pathways offers a viable approach for prevention or delay of preterm labor.

Limitations of the study

Our data support a mechanism for allosteric regulation of EP2 signaling via associations with the Gαi/o-coupled OTR; however, a current limitation of this study is demonstrating that directly disrupting specific heteromer complexes in primary myocytes prevents EP2/OTR crosstalk. However, the complexity in heteromer interfaces identified by PD-PALM with molecular modeling, as observed with other GPCRs (Jonas et al., 2015), makes this a challenging complex to disrupt. However, the evidence for functional interaction between OTR and EP2 presented here unveils potential targets for therapeutic exploitation. By focusing on pregnant human tissue obtained before or during labor, our findings provide human models of GPCR signaling in labor; however, it also has inherent limitations of the *in vitro* system. While future studies could progress to animal preclinical *in vivo* models of preterm labor, these may offer additional limitations and challenges in the translation to human pregnancy and labor (Carter, 2020; Nielsen et al., 2016).

STAR★METHODS

Detailed methods are provided in the online version of this paper and include the following:

- KEY RESOURCES TABLE
- RESOURCE AVAILABILITY
  - Lead contact
  - Materials availability
  - Data and code availability
- EXPERIMENTAL MODEL AND SUBJECT DETAILS
  - Primary myometrial cell cultures
  - Myometrial tissue dissociation, culture and transfection
  - Cell lines

CellPress
OPEN ACCESS

- METHOD DETAILS
  - Experimental design
  - Reagents and plasmids
  - RNA isolation and sequencing
  - RNA-seq data analysis
  - Photo-activated localization microscopy with photoactivatable dyes (PD-PALM)
  - Structural modeling
  - Measurement of intracellular Ca^{2+}
  - Proximity ligation assay
  - Intracellular cAMP assay
  - Bioluminescence resonance energy transfer (BRET)
  - Western Blot
  - Measurement of secreted PGE2 and IL-6
- QUANTIFICATION AND STATISTICAL ANALYSIS

SUPPLEMENTAL INFORMATION

Supplemental information can be found online at https://doi.org/10.1016/j.celrep.2022.111318.

ACKNOWLEDGMENTS

We would like to thank all patients and staff at Queen Charlotte's Hospital and Chelsea and Westminster Hospital for providing and assisting with the collection of samples. We would like to thank Drs. Andreas Bruckbauer and Stephen Rothery at the Facility for Imaging of Light Microscopy (FILM) at Imperial College London for technical support with PD-PALM. We would like to acknowledge the use of Servier Medical Art image bank to aid in the creation of the graphical abstract. We would like to acknowledge the following funding bodies for making this work possible: Biotechnology and Biological Sciences (BB/V006142/1, A.C.H.); Genesis Research Trust P65251 (A.C.H.); Imperial NIHR Biomedical Research Centre (P.R.B.); Society for Endocrinology Early Career Grant (S. Khanjani); Japan Agency for Medical Research and Development PRIME 19gm5910013 (A.I.); Japan Agency for Medical Research and Development LEAP 19gm0010004 (A.I.); Japan Agency for Medical Research and Development BINDS JP20am0101095 (A.I.); Japan Society for the Promotion of Science KAKENHI 21H04791 (A.I.); Japan Science and Technology Agency (JST) JST Moonshot Research and Development Program JPMJMS2023 (A.I.); and The Italian Ministry for Education, University and Research PRIN2017 2017R5ZE2C (F.F.).

AUTHOR CONTRIBUTIONS

A.C.H. and P.R.B. conceived the study and, with A.R.W., designed experiments. C.B.L. carried out the experimental studies on early and late labor samples under the supervision of A.C.H. C.S. undertook the PTX studies on IL-6 and COX-2 under supervision of A.C.H. A.R.W. performed all other experiments and analyzed all datasets under supervision of A.C.H. and P.R.B. S. Khanjani consented patients and provided all early and late labor myometrial samples, and V.T. consented patients and, with S. Khanjani, provided all non-laboring myometrial cultures and provided reagents. S. Kundu, D.A.M., Y.S.L., and R.M. performed RNA-seq preparation and analysis. A.I. designed and generated the $\Delta G\alpha q/11$ cells. F.F. performed all molecular modeling studies. D.F.W. provided a series of compounds from which PGN9856i was selected, by consensus, for study. A.R.W., A.C.H., P.R.B., and D.F.W. wrote the manuscript, and all authors contributed to and approved the final version.

DECLARATION OF INTERESTS

An international patent application for the method of use of PGN9856i in preterm labor described in this manuscript has been filed (PCT/GB2021/051,971) on behalf of A.R.W., P.R.B., and A.C.H. by Imperial College London. The remaining authors declare no competing interests.

Received: January 25, 2022
Revised: June 21, 2022
Accepted: August 16, 2022
Published: September 6, 2022

REFERENCES

Albizu, L., Cottet, M., Kralikova, M., Stoev, S., Seyer, R., Brabet, I., Roux, T., Bazin, H., Bourrier, E., Lamarque, L., et al. (2010). Time-resolved FRET between GPCR ligands reveals oligomers in native tissues. Nat. Chem. Biol. *6*, 587–594. https://doi.org/10.1038/nchembio.396.

Ayoub, M.A., and Pfleger, K.D.G. (2010). Recent advances in bioluminescence resonance energy transfer technologies to study GPCR heteromerization. Curr. Opin. Pharmacol. *10*, 44–52. https://doi.org/10.1016/j.coph.2009.09.012.

Balenga, N.A., Martínez-Pinilla, E., Kargl, J., Schröder, R., Peinhaupt, M., Platzer, W., Bálint, Z., Zamarbide, M., Dopeso-Reyes, I.G., Ricobaraza, A., et al. (2014). Heteromerization of GPR55 and cannabinoid CB2 receptors modulates signalling. Br. J. Pharmacol. *171*, 5387–5406. https://doi.org/10.1111/bph.12850.

Bertrand, J.A., Woodward, D.F., Sherwood, J.M., Wang, J.W., and Overby, D.R. (2020). The role of EP 2 receptors in mediating the ultra- lasting intraocular pressure reduction by JV-GL[1]. Br. J. Ophthalmol. *105*, 1610–1616. https://doi.org/10.1136/bjophthalmol-2020-317762.

Buenaventura, T., Bitsi, S., Laughlin, W.E., Burgoyne, T., Lyu, Z., Oqua, A.I., Norman, H., McGlone, E.R., Klymchenko, A.S., Corrêa, I.R., Jr., et al. (2019). Agonist-induced membrane nanodomain clustering drives GLP-1 receptor responses in pancreatic beta cells. PLoS Biol. *17*, e3000097. https://doi.org/10.1371/journal.pbio.3000097.

Calebiro, D., Rieken, F., Wagner, J., Sungkaworn, T., Zabel, U., Borzi, A., Cocucci, E., Zürn, A., and Lohse, M.J. (2013). Single-molecule analysis of fluorescently labeled G-protein-coupled receptors reveals complexes with distinct dynamics and organization. Proc. Natl. Acad. Sci. USA *110*, 743–748. https://doi.org/10.1073/pnas.1205798110.

Callén, L., Moreno, E., Barroso-Chinea, P., Moreno-Delgado, D., Cortés, A., Mallol, J., Casadó, V., Lanciego, J.L., Franco, R., Lluis, C., et al. (2012). Cannabinoid receptors CB 1 and CB 2 form functional heteromers in brain. J. Biol. Chem. *287*, 20851–20865. https://doi.org/10.1074/jbc.M111.335273.

Carter, A.M. (2020). Animal models of human pregnancy and placentation: alternatives to the mouse. Reproduction *160*, R129–R143. https://doi.org/10.1530/REP-20-0354.

Casarini, L., Lazzaretti, C., Paradiso, E., Limoncella, S., Riccetti, L., Sperduti, S., Melli, B., Marcozzi, S., Anzivino, C., Sayers, N.S., et al. (2020). Membrane estrogen receptor (GPER) and follicle-stimulating hormone receptor (FSHR) heteromeric complexes promote human ovarian follicle survival. iScience *23*, 101812. https://doi.org/10.1016/j.isci.2020.101812.

Casciari, D., Seeber, M., and Fanelli, F. (2006). Quaternary structure predictions of transmembrane proteins starting from the monomer: a docking-based approach. BMC Bioinf. *7*, 340. https://doi.org/10.1186/1471-2105-7-340.

Chawanpaiboon, S., Vogel, J.P., Moller, A.B., Lumbiganon, P., Petzold, M., Hogan, D., Landoulsi, S., Jampathong, N., Kongwattanakul, K., Laopaiboon, M., et al. (2019). Global, regional, and national estimates of levels of preterm birth in 2014: a systematic review and modelling analysis. Lancet Global Health *7*, e37–e46. https://doi.org/10.1016/S2214-109X(18)30451-0.

Chen, R., Li, L., and Weng, Z. (2003). ZDOCK: an initial-stage protein-docking algorithm. Proteins *52*, 80–87. https://doi.org/10.1002/prot.10389.

Coleman, R.A., Woodrooffe, A.J., Clark, K.L., Toris, C.B., Fan, S., Wang, J.W., Woodward, D.F., David Woodward, C.F., Coleman, R.A., Woodrooffe, A.J., et al. (2019). The affinity, intrinsic activity and selectivity of a structurally novel EP 2 receptor agonist at human prostanoid receptors. Br. J. Pharmacol. *176*, 687–698. https://doi.org/10.1111/bph.14525.

Crowther, C.A., Brown, J., Mckinlay, C.J.D., and Middleton, P. (2014). Magnesium sulphate for preventing preterm birth in threatened preterm labour.

Cochrane Database Syst. Rev. *2014*, CD001060. https://doi.org/10.1002/14651858.CD001060.PUB2.

de la Mora, M.P., Pérez-Carrera, D., Crespo-Ramírez, M., Tarakanov, A., Fuxe, K., and Borroto-Escuela, D.O. (2016). Signaling in dopamine D2 receptor-oxytocin receptor heterocomplexes and its relevance for the anxiolytic effects of dopamine and oxytocin interactions in the amygdala of the rat. Biochim. Biophys. Acta *1862*, 2075–2085. https://doi.org/10.1016/j.bbadis.2016.07.004.

Dobin, A., Davis, C.A., Schlesinger, F., Drenkow, J., Zaleski, C., Jha, S., Batut, P., Chaisson, M., and Gingeras, T.R. (2013). STAR: ultrafast universal RNA-seq aligner. Bioinformatics *29*, 15–21.

Europe-Finner, G.N., Phaneuf, S., Tolkovsky, A.M., Watson, S.P., and López Bernal, A. (1994). Down-regulation of G alpha s in human myometrium in term and preterm labor: a mechanism for parturition. J. Clin. Endocrinol. Metab. *79*, 1835–1839. https://doi.org/10.1210/jcem.79.6.7989491.

Fanelli, F., Seeber, M., Felline, A., Casciari, D., and Raimondi, F. (2013). Quaternary structure predictions and structural communication features of GPCR dimers. In Progress in Molecular Biology and Translational Science (Elsevier B.V.), pp. 105–142.

Ferrada, C., Moreno, E., Casadó, V., Bongers, G., Cortés, A., Mallol, J., Canela, E.I., Leurs, R., Ferré, S., Lluís, C., and Franco, R. (2009). Marked changes in signal transduction upon heteromerization of dopamine D 1 and histamine H 3 receptors. Br. J. Pharmacol. *157*, 64–75. https://doi.org/10.1111/j.1476-5381.2009.00152.x.

Grundmann, M., Merten, N., Malfacini, D., Inoue, A., Preis, P., Simon, K., Rüttiger, N., Ziegler, N., Benkel, T., Schmitt, N.K., et al. (2018). Lack of beta-arrestin signaling in the absence of active G proteins. Nat. Commun. *9*, 341–416. https://doi.org/10.1038/s41467-017-02661-3.

Hanyaloglu, A.C., McCullagh, E., and Von Zastrow, M. (2005). Essential role of Hrs in a recycling mechanism mediating functional resensitization of cell signaling. EMBO J. *24*, 2265–2283. https://doi.org/10.1038/sj.emboj.7600688.

Hauser, A.S., Attwood, M.M., Rask-Andersen, M., Schiöth, H.B., and Gloriam, D.E. (2017). Trends in GPCR drug discovery: new agents, targets and indications. Nat. Rev. Drug Discov. *16*, 829–842. https://doi.org/10.1038/nrd.2017.178.

Heyer, L.J., Kruglyak, S., and Yooseph, S. (1999). Exploring expression data identification and analysis of coexpressed genes. Genome Res. *9*, 1106–1115. https://doi.org/10.1101/gr.9.11.1106.

Jonas, K.C., Fanelli, F., Huhtaniemi, I.T., and Hanyaloglu, A.C. (2015). Single molecule analysis of functionally asymmetric G protein-coupled receptor (GPCR) oligomers reveals diverse spatial and structural assemblies. J. Biol. Chem. *290*, 3875–3892. https://doi.org/10.1074/jbc.M114.622498.

Jonas, K.C., Huhtaniemi, I., and Hanyaloglu, A.C. (2016). Single-molecule resolution of G protein-coupled receptor (GPCR) complexes. Methods Cell Biol. *132*, 55–72. https://doi.org/10.1016/BS.MCB.2015.11.005.

Jonas, K.C., Chen, S., Virta, M., Mora, J., Franks, S., Huhtaniemi, I., and Hanyaloglu, A.C. (2018). Temporal reprogramming of calcium signalling via crosstalk of gonadotrophin receptors that associate as functionally asymmetric heteromers. Sci. Rep. *8*, 2239. https://doi.org/10.1038/s41598-018-20722-5.

Kandola, M.K., Sykes, L., Lee, Y.S., Johnson, M.R., Hanyaloglu, A.C., and Bennett, P.R. (2014). EP2 receptor activates dual g protein signaling pathways that mediate contrasting proinflammatory and relaxatory responses in term pregnant human myometrium. Endocrinology *155*, 605–617. https://doi.org/10.1210/en.2013-1761.

Kang, Y., Kuybeda, O., De Waal, P.W., Mukherjee, S., Van Eps, N., Dutka, P., Zhou, X.E., Bartesaghi, A., Erramilli, S., Morizumi, T., et al. (2018). Cryo-EM structure of human rhodopsin bound to an inhibitory G protein. Nature *558*, 553–558. https://doi.org/10.1038/s41586-018-0215-y.

Karteris, E., Zervou, S., Pang, Y., Dong, J., Hillhouse, E.W., Randeva, H.S., and Thomas, P. (2006). Progesterone signaling in human myometrium through two novel membrane G protein-coupled receptors: potential role in functional progesterone withdrawal at term. Mol. Endocrinol. *20*, 1519–1534. https://doi.org/10.1210/ME.2005-0243.

Kasai, R.S., Suzuki, K.G.N., Prossnitz, E.R., Koyama-Honda, I., Nakada, C., Fujiwara, T.K., and Kusumi, A. (2011). Full characterization of GPCR monomer-dimer dynamic equilibrium by single molecule imaging. J. Cell Biol. *192*, 463–480. https://doi.org/10.1083/jcb.201009128.

Kim, S.H., MacIntyre, D.A., Firmino Da Silva, M., Blanks, A.M., Lee, Y.S., Thornton, S., Bennett, P.R., and Terzidou, V. (2015). Oxytocin activates NF-κB-mediated inflammatory pathways in human gestational tissues. Mol. Cell. Endocrinol. *403*, 64–77. https://doi.org/10.1016/j.mce.2014.11.008.

Kim, S.H., MacIntyre, D.A., Hanyaloglu, A.C., Blanks, A.M., Thornton, S., Bennett, P.R., and Terzidou, V. (2016). The oxytocin receptor antagonist, Atosiban, activates pro-inflammatory pathways in human amnion via Gαi signalling. Mol. Cell. Endocrinol. *420*, 11–23. https://doi.org/10.1016/j.mce.2015.11.012.

Kim, S.H., Riaposova, L., Ahmed, H., Pohl, O., Chollet, A., Gotteland, J.P., Hanyaloglu, A., Bennett, P.R., and Terzidou, V. (2019). Oxytocin receptor antagonists, atosiban and nolasiban, inhibit prostaglandin F 2α -induced contractions and inflammatory responses in human myometrium. Sci. Rep. *9*, 5792. https://doi.org/10.1038/s41598-019-42181-2.

Lamont, R.F., and Jørgensen, J.S. (2019). Safety and efficacy of tocolytics for the treatment of spontaneous preterm labour. Curr. Pharmaceut. Des. *25*, 577–592. https://doi.org/10.2174/1381612825666190329124214.

Lamont, C.D., Jørgensen, J.S., and Lamont, R.F. (2016). The safety of tocolytics used for the inhibition of preterm labour. Expet Opin. Drug Saf. *15*, 1163–1173. https://doi.org/10.1080/14740338.2016.1187128.

Liao, Y., Smyth, G.K., and Shi, W. (2014). featureCounts: an efficient general purpose program for assigning sequence reads to genomic features. Bioinformatics *30*, 923–930.

Love, M.I., Huber, W., and Anders, S. (2014). Moderated estimation of fold change and dispersion for RNA-seq data with DESeq2. Genome Biol *15*, 1–21.

Meyerowitz, J.G., Robertson, M.J., Barros-Álvarez, X., Panova, O., Nwokonko, R.M., Gao, Y., and Skiniotis, G. (2022). The oxytocin signaling complex reveals a molecular switch for cation dependence. Nat. Struct. Mol. Biol. *29*, 274–281. https://doi.org/10.1038/s41594-022-00728-4.

Nials, A.T., Vardey, C.J., Denyer, L.H., Thomas, M., Sparrow, S.J., Shepherd, G.D., and Coleman, R.A. (1993). AH13205, A selective prostanoid EP2-receptor agonist. Cardiovasc. Drug Rev. *11*, 165–179.

Nielsen, B.W., Bonney, E.A., Pearce, B.D., Donahue, L.R., and Sarkar, I.N.; Preterm Birth International Collaborative PREBIC (2016). A cross-species analysis of animal models for the investigation of preterm birth mechanisms. Reprod. Sci. *23*, 482–491. https://doi.org/10.1177/1933719115604729.

Romero-Fernandez, W., Borroto-Escuela, D.O., Agnati, L.F., and Fuxe, K. (2013). Evidence for the existence of dopamine d2-oxytocin receptor heteromers in the ventral and dorsal striatum with facilitatory receptor-receptor interactions. Mol. Psychiatr. *18*, 849–850. https://doi.org/10.1038/mp.2012.103.

Rozenfeld, R., Gupta, A., Gagnidze, K., Lim, M.P., Gomes, I., Lee-Ramos, D., Nieto, N., and Devi, L.A. (2011). AT1R-CB1 R heteromerization reveals a new mechanism for the pathogenic properties of angiotensin II. EMBO J. *30*, 2350–2363. https://doi.org/10.1038/emboj.2011.139.

Šali, A., and Blundell, T.L. (1993). Comparative protein modelling by satisfaction of spatial restraints. J. Mol. Biol. *234*, 779–815. https://doi.org/10.1006/jmbi.1993.1626.

Seeber, M., Felline, A., Raimondi, F., Muff, S., Friedman, R., Rao, F., Caflisch, A., and Fanelli, F. (2011). Wordom: a user-friendly program for the analysis of molecular structures, trajectories, and free energy surfaces. J. Comput. Chem. *32*, 1183–1194. https://doi.org/10.1002/jcc.21688.

Slater, D.M., Astle, S., Woodcock, N., Chivers, J.E., de Wit, N.C.J., Thornton, S., Vatish, M., and Newton, R. (2006). Anti-inflammatory and relaxatory effects of prostaglandin E2 in myometrial smooth muscle. Mol. Hum. Reprod. *12*, 89–97. https://doi.org/10.1093/MOLEHR/GAL005.

Stanfield, Z., Lai, P.F., Lei, K., Johnson, M.R., Blanks, A.M., Romero, R., Chance, M.R., Mesiano, S., and Koyutürk, M. (2019). Myometrial

transcriptional signatures of human parturition. Front. Genet. *10*, 185. https://doi.org/10.3389/FGENE.2019.00185/BIBTEX.

Sugimoto, Y., and Narumiya, S. (2007). Prostaglandin E receptors * downloaded from. J. Biol. Chem. *282*, 11613–11617. https://doi.org/10.1074/jbc.R600038200.

Terrillon, S., Durroux, T., Mouillac, B., Breit, A., Ayoub, M.A., Taulan, M., Jockers, R., Barberis, C., and Bouvier, M. (2003). Oxytocin and vasopressin V1a and V2 receptors form constitutive homo- and heterodimers during biosynthesis. Mol. Endocrinol. *17*, 677–691. https://doi.org/10.1210/me.2002-0222.

Terzidou, V., Sooranna, S.R., Kim, L.U., Thornton, S., Bennett, P.R., and Johnson, M.R. (2005). Mechanical stretch up-regulates the human oxytocin receptor in primary human uterine myocytes. J. Clin. Endocrinol. Metab. *90*, 237–246. https://doi.org/10.1210/jc.2004-0277.

Walani, S.R. (2020). Global burden of preterm birth. Int. J. Gynaecol. Obstet. *150*, 31–33. https://doi.org/10.1002/ijgo.13195.

Woodward, D.F., Wang, J.W., Coleman, R.A., Woodrooffe, A.J., Clark, K.L., Stamer, W.D., Tao, G., Fan, S., and Toris, C.B. (2019). A highly effective and ultra-long-acting anti-glaucoma drug , with a novel periorbital delivery method. J. Ocul. Pharmacol. Therapeut. *35*, 265–277. https://doi.org/10.1089/jop.2018.0126.

STAR★METHODS

KEY RESOURCES TABLE

REAGENT or RESOURCE	SOURCE	IDENTIFIER
Antibodies		
Anti-Cox-2 Antibody (C-20): sc-1745	Santa Cruz	SC-1745; RRID:AB_631309
Anti-GAPDH	Millipore	AB2302; RRID:AB_10615768
Anti-HA.11 Epitope Tag	Biolegend	901502; RRID:AB_2565007
HRP- conjugated IgG anti-Goat	Santa Cruz	SC- 2354; RRID:AB_628490
HRP- conjugated IgG anti-Mouse	Thermo Fisher Scientific	626520; RRID:AB_2533947
Anti-EP2 antibody (clone H-75)	Santa Cruz	SC-20675; RRID:AB_641256
Anti-OTR goat antibody (clone N-19)	Santa Cruz	SC-8103 RRID:AB_2157759
Biological samples		
Primary human myometrium	Chelsea and Westminster Hospital and Queen Charlotte's and Chelsea Hospital, London, UK	N/A
Chemicals, peptides, and recombinant proteins		
CAGE 500	Abberior	CA500
CAGE 552	Abberior	CA552
Critical commercial assays		
cAMP dynamic kit	Cisbio	62AM4PEB
Fluo-4 direct calcium assay	Invitrogen	F10441
IL-6 ELISA Deluxe	Biolegend	430504
PGE2 ELISA	Enzo Life Sciences	ADI-900-001
Duolink *In situ* Orange Starter kit Goat/Rabbit	Sigma Aldrich	DUO92106
Lipofectamine 2000	Invitrogen	11668019
cAMP competitive immunoassay	Assay Designs	ADI-901-066
Deposited data		
RNA-seq data	This paper	European Nucleotide Archive: PRJEB52170; https://www.ebi.ac.uk/ena/browser/view/PRJEB52170
Experimental models: cell lines		
ΔGαq/11 HEK 293	Asuka Inoue	N/A
HEK 293	ATCC	CRL-1573; RRID:CVCL_0045
Recombinant DNA		
Plasmid: EP2-FLAG	This manuscript	N/A
Plasmid: OTR-HA	Marta Busnelli	N/A
Plasmid: EP2-Rluc8	This manuscript	N/A
Plasmid: OTR-Rluc	Marta Busnelli	N/A
Plasmid: EP2-HA	Barrie Ashby	N/A
Plasmid: OTR-Venus	This manuscript	N/A

RESOURCE AVAILABILITY

Lead contact

Further information and requests for resources and reagents should be directed to and will be fulfilled by the lead contact, Aylin Hanyaloglu (a.hanyaloglu@imperial.ac.uk).

Materials availability

FLAG-EP2, HA-OTR, EP2-Rluc8 and OTR-Venus are available from A.C.H. under a materials transfer agreement with Imperial College London

Data and code availability

- Sequencing data have been deposited at European Nucleotide Archive (ENA) repository (https://www.ebi.ac.uk/ena) and are publicly available as of the date of publication. Study accession number is listed in the key resources table and run accession numbers are listed in Table S1. All data reported in this paper will be shared by the lead contact upon request.
- This paper does not report original code
- Any additional information required to reanalyze the data reported in this paper is available from the lead contact upon request

EXPERIMENTAL MODEL AND SUBJECT DETAILS

Primary myometrial cell cultures

Primary myometrial tissue was obtained from women provided with informed written consent prior to participation, with approval from the Riverside Research Ethics Committee (REC 3357, 1997-5089) and London Harrow Research Ethics Committee (REC 19/LO/1657). Experiments were carried out under the committee's guidelines and recommendations.

Myometrial tissue was acquired from women of reproductive age at Chelsea and Westminster Hospital or Queen Charlotte's and Chelsea Hospital, London, UK. Tissues were obtained from term (38^{+0} - 40 weeks gestation) pregnant women undergoing elective caesarean from the upper margin of the incision made at the lower segment of the uterus, before or after the onset of labor (with or without labor induction using intravenous OT (syntocinon)). Samples taken after the onset of labor were divided into early labor (cervical dilution <3 cm) and late labor (cervical dilution > 3 cm). Tissue was only taken from uncomplicated, singleton pregnancies.

Myometrial tissue dissociation, culture and transfection

Tissue was stored at 4°C in phosphate buffered saline until fine dissection with scalpels and dissociation at 37°C for 1h in a sterile-filtered mix of Dulbecco's modified Eagle's medium (DMEM)(Sigma Aldrich)/Ham's F-12 Nutrient Mixture (Sigma Aldrich) and serum-free DMEM (Sigma Aldrich) (1:1 v/v) containing collagenase 1A (Sigma Aldrich, 1 mg/ml), collagenase X (Sigma Aldrich, 1mg/ml) and bovine serum albumin (Sigma Aldrich, 2 mg/mL). Dissociation was ended using DMEM 10% fetal bovine serum (Sigma Aldrich) and cell suspension was obtained using a 40 μm cell strainer before centrifugation at 3000 rpm for 5 min. Cell pellet was resuspended in DMEM containing 10% fetal bovine serum and 100 U/mL penicillin-streptomycin (Sigma Aldrich) and cultured in 75cm flasks at 37°C in 95% air and 5% CO2. Cells at 95% confluency were passaged using 0.25% trypsin with 0.02% EDTA in phosphate-buffered saline. Myocytes cultured from tissue following labor onset were used at passage 0, those from non-laboring women were used until passage 5. Myometrial cells were transfected using Lipofectamine 2000 (Invitrogen) in DMEM containing 10% FBS without antibiotics for 72h.

Cell lines

HEK 293 and ΔGαq/11 cell culture and transfection

HEK 293 cells (female) and ΔGαq/11 HEK 293 cells (female) were maintained in DMEM containing 10% fetal bovine serum and 100 U/mL penicillin-streptomycin and cultured in 75cm flasks at 37°C in 95% air and 5% CO2. Cells at 90% confluency were passaged using 0.25% trypsin with 0.02% EDTA in phosphate-buffered saline. HEK 293 and ΔGαq/11 HEK 293 cells were transfected using Lipofectamine 2000 (Invitrogen) in DMEM containing 10% FBS without antibiotics for 72h.

METHOD DETAILS

Experimental design

The goals of this study were to delineate the functional relationship between OT/OTR and EP2 signaling in human term pregnant myometrium during labor and to define the preclinical relevance of targeting EP2 for tocolytic purposes. The hypothesis was built on our previous findings that EP2 can signal via Gαs and Gαq/11 pathways in pregnant myometrium prior to labor, allowing it to activate pro- and anti-labor pathways. However, following labor onset only the pro-labor pathway was maintained. This objective was accomplished by (i) assessing EP2 signaling changes in the myometrium before and after distinct stages of labor (early vs late, spontaneous vs induced). (ii) mechanistically dissecting the functional relationship between OT signaling and EP2 in non-laboring pregnant myometrium and validating these changes in laboring samples (iii) using energy-transfer, proximity ligation, a super-resolution imaging technique and molecular modeling to assess the existence of EP2-OTR heteromers. Experiments in cell lines were performed independently a minimum of three times unless stated otherwise in the figure legend. For *in vitro* treatment studies on primary myocyte cultures, there have been no prior studies employing the distinct labor samples (induced/spontaneous, early/late) for functional signaling studies, thus we employed n = 4–5 in each patient group based on our prior *in vitro* GPCR signaling studies in primary myocytes (Kandola et al., 2014; Kim et al., 2015, 2019; Terzidou et al., 2005).

Reagents and plasmids

Antibodies used were mouse anti-GAPDH (Millipore); goat anti-COX-2 (Santa-Cruz); mouse anti-goat (Santa-Cruz) and goat anti-mouse (Thermo Fisher Scientific) horseradish peroxidase (HRP). The inhibitors used were PTX (pertussis toxin) (Tocris) at 200ng/mL (16-hour pre-treatment) and IBMX (3-Isobutyl-1-methylxanthine) (Sigma) used at 0.5mM (5-minute pre-treatment). Butaprost, AH-13205 and isoproterenol were from Sigma-Aldrich and were used at 10 μM. Oxytocin was from Sigma and used at 100 nM. PGN9856i was an unrestricted gift from Allergan Inc. and was used at 100 nM.

HA-OTR and OTR-Rluc were kindly provided by Marta Busnelli (Institute of Neuroscience, Milan, Italy) and were used to construct OTR-Venus for BRET studies. OTR was PCR amplified without its stop codon and ligated into HindIII and KpnI recognition sites into a pcDNA3.1 vector containing Venus YFP. HA-EP2 was kindly gifted from Barrie Ashby (Temple University School of Medicine, USA) and was used to construct EP2-Rluc8 for BRET studies. EP2 was PCR amplified without its stop codon and ligated into Nhe1 and Not1 recognition sites in a pcDNA3.1 vector containing Rluc8 to form EP2-Rluc8. FLAG-LHR was kindly gifted by Ilpo Huhtaniemi (Imperial College London, UK) and was used alongside HA-EP2 to construct FLAG-EP2. EP2 was amplified from the HA-EP2 plasmid by PCR and ligated into EcoRV and XbaI sites into a FLAG-LHR/pcDNA3.1 plasmid and digested to remove the LHR sequence using with AfeI and XbaI.

RNA isolation and sequencing

Frozen myometrium tissue (25 ug/sample) was homogenised in liquid nitrogen and RNA was extracted using the NucleoSpin® miRNA kit (Macherey-Nagel) with DNase treatment following manufacturer's instructions. RNA quality was assessed for all samples using an RNA 6000 Nano Kit and Bioanalyzer (Agilent). Only samples with an RNA integrity number (RIN) > 8 were used for RNA-Seq library preparation. cDNA libraries with an insert length of 300 bp were prepared from 2 μg of purified large RNA fraction (>200 nucleotides) using a TruSeq Stranded mRNA Sample preparation kit (Illumina). Samples were enriched for poly-A mRNA using oligo-dT-coated magnetic beads. Following purification, mRNA was randomly fragmented at 94°C for 8 min to obtain fragments of around 200 bp while minimizing bias at the 3′ end of transcripts. First-strand complementary DNA synthesis was performed using random primers (Illumina) and SuperScript II Reverse-Transcriptase (Invitrogen) followed by second strand synthesis with RNaseH and DNA polymerase I (Illumina). Adapters provided were used to tag each sample. Compatibility between adapters was checked with Illumina Experiment Manager to allow subsequent pooling of seven samples in each lane during sequencing. cDNA libraries were amplified by 10 cycles of PCR. The quality of each library was evaluated on a 2100 Bioanalyzer (Agilent) followed by paired-end sequencing (2 × 125 bp) on an Illumina HiSeq 2000. Sequencing data has been deposited on the European Nucleotide Archive (ENA) repository (https://www.ebi.ac.uk/ena). Accession numbers are listed in the Key Resources Table and in Supplemental Materials, Table S1.

RNA-seq data analysis

A total of 1,026,519,794 reads were generated across the 25 samples (mean 41060792 reads/sample). Read quality was assessed using FastQC (https://www.bioinformatics.babraham.ac.uk/projects/fastqc/) and trimming performed using TrimGalore (https://www.bioinformatics.babraham.ac.uk/projects/trim_galore/). Reads were aligned to the GRCh38 reference human genome using the STAR RNA-seq aligner (Dobin et al., 2013) with between 91.2 and 94.6% of reads mapped to the human genome reference. We used featureCounts (Liao et al., 2014) to obtain read counts per gene. Finally, counts were transformed using the variance stabilizing transformation in DESeq2 (Love et al., 2014) .

Photo-activated localization microscopy with photoactivatable dyes (PD-PALM)

HEK 293 cells stably expressing FLAG-EP2 at 85–90% confluence were transfected with HA-OTR and plated onto 35mm dishes (Mattek) with 14 mm × 1.5 mm glass coverslips. Anti-HA.11 (Biolegend) and anti-FLAG (Sigma Aldrich) primary antibodies were labelled with CAGE 552 and CAGE 500 photoswitchable dyes as per manufacturer's instructions (Abberior) and the degree of labelling efficiency was determined to be 1.058 and 0.991 dye molecules per antibody for CAGE 552 and CAGE 500, respectively. Cells were labeled live with CAGE 500 and CAGE 552 conjugated antibodies for 30 min at 37°C, washed 3 times in PBS/Ca^{2+} and fixed in 4% paraformaldehyde (Sigma Aldrich) with 0.2% Glutaraldehyde (Sigma Aldrich) for 30 min. Cells were washed and maintained in PBS/Ca^{2+} until imaging. All labelling and steps between labelling and imaging were carried out in the dark to prevent activation of CAGE antibodies.

Images were acquired in the TIRF plane with a Zeiss Elyra PS1 super-resolution microscope using a 1.45 numerical aperture ×100 oil immersion objective. The microscope was contained in a draft-proof enclosure on a vibration isolation table and kept at a constant temperature of 25°C. Photo-conversion of CAGE dyes was achieved using a polychrome light source at 390 nm which were simultaneously activated and photo-bleached by 491nm laser for CAGE 500 dyes and 561nm laser for CAGE 552. Laser lines were switched on at least 30 min before imaging to allow stabilization of the system, minimizing drift during experiments. Images were captured using ZEN software with a 30 ms exposure time. Localization analysis of receptors imaged in 491 and 561nm channels was achieved using a Fiji plugin, QuickPALM. Two non-overlapping images of 7 μm × 7 μm areas were taken for each cell images. This was done within cell borders to prevent bias from cell edges. The areas were analyzed with the following QuickPALM parameters: a signal to noise ratio of 7 and a full-half width maximum of 5, generating a table of x,y co-ordinates for the localized particles. Particles within 10 nm of each other in the same channel were discounted to prevent overestimation of associated receptors,

CellPress
OPEN ACCESS

resulting in a localization precision of 20 nm. To determine the number of associated receptor molecules from the x,y co-ordinates a custom java app was used: PD-Interpreter (Jonas et al., 2015, 2016, 2018). Using a search radius of 50 nm, a second order Getis Franklin neighborhood analysis was conducted, to determine the number of receptor associations within channels (homomeric associations), and across channels (heteromeric associations). Once an associating group of molecules was found, the particles within it were excluded from future searches, to prevent double counting.

Structural modeling

At the time of analysis, no crystallographic structure was available for EP2. The structural model of the inactive state of the receptor was achieved by comparative modeling (by the Modeller software (Šali and Blundell, 1993), using a chimeric structure between EP3 (PDB: 6M9T) and EP4 (PDB: 5YHL). The chimera is characterized by the region H2-H5 (H stands for helix) from EP3 and H1,H6-H8 from EP4. The first intracellular loop (I1) was not included. H8 was rotated to an orientation shared with the majority of inactive-state GPCRs. One-hundred models were built by randomizing all the Cartesian coordinates of standard residues in the initial model. The best model according to quality checks was subjected to application of rotamer libraries to those side chains in non-allowed conformation. The structural model of OTR in its inactive state was the crystal structure encoded as 6TPK, following completion of H8. The approximate active-state structure of OTR was achieved by replacing the stretch 268–289 of H6 in the crystal structure with the stretch extracted from a structural model of EP2 achieved by using the crystal structure of the μ-opioid receptor (PDB: 5C1M) as a template. The structural models of EP2 and OTR finally employed for docking simulations lack N- and C-terms, as well as I1 and I3.

Prediction of likely architectures of EP2-OTR heteromers followed a computational approach developed for quaternary structure predictions of transmembrane α-helical proteins, defined as a FiPD-based approach (Casciari et al., 2006; Fanelli et al., 2013). It consists in rigid-body docking using a version of the ZDOCK program devoid of desolvation as a component of the docking score (v2.1) (Chen et al., 2003). A rotational sampling interval of 6° was set (i.e. dense sampling) and the best 4000 solutions were retained and ranked according to the ZDOCK score. Such solutions were filtered according to the "membrane topology" filter (by using the FiPD software (Casciari et al., 2006)), which discards all those solutions that violate the membrane topology requirements. The membrane topology filter, indeed, discards all the solutions characterized by a deviation angle from the original z-axis, i.e., tilt angle, and a displacement of the geometrical centre along the z-axis, i.e., z-offset, above defined threshold values, which were 0.4 radians and 6.0 Å, respectively. The filtered solutions from each run were merged with the target protein, leading to an equivalent number of dimers (in the first step) or trimers or tetramers that were clustered using a C_α-RMSD threshold of 3.0 Å for each pair of superimposed dimers/higher order oligomers. All the amino acid residues in the complexes were included in C_α-RMSD calculations. Cluster analysis was based on a QT-like clustering algorithm (Heyer et al., 1999) implemented both in the FiPD and Wordom software (Casciari et al., 2006; Seeber et al., 2011). Since the filtering cut-offs of the membrane topology parameters are intentionally quite permissive, inspection of the cluster centres (i.e., the solutions with the highest number of neighbours in each cluster) served as a final filter to discard remaining false positives, thereby leading to a dramatic reduction of the reliable solutions. The best scored docking solutions from the most populated and reliable clusters were finally considered. Cluster reliability was based on the MemTop score, accounting for the goodness of the membrane topology. Simulations started from homo- and hetero-dimerization and ended to prediction of heterotetramers.

Measurement of intracellular Ca^{2+}

Myometrial cells were seeded onto 35 mm dishes (Mattek) with 14 mm × 1.5 mm glass coverslips. For PTX (200 ng/mL) (Tocris) conditions, WT HEK 293 cells were pretreated for 15h, while experiments employing ΔGαq/11 HEK 293 cells, PTX was added for 3h. Cells were incubated with Fluo-4AM Ca^{2+} indicator (ThermoFisher) as per manufacturer's instructions for 30 min at 37°C and 30 min at room temperature. Time-series images were acquired using a Leica SP5 confocal microscope every 1.2 s, Leica LAS AF image acquisition software and a 488nm excitation laser. HEK cells and myometrial cells were imaged using a 10× or 20× dry objective, respectively. Raw files were analyzed using Fiji Time series analyzer plugin to quantify the maximal fluorescent intensity in at least 30 cells per sample, which was then averaged across cells in each condition.

Proximity ligation assay

Anti-EP2 rabbit antibody (H-75) and anti-OTR goat antibody (N-19) (Santa Cruz Biotechnology) that recognize the N-terminus of the human EP2 or OTR was used at 1:50 dilution. The EP2 and OTR interactions in primary myocyte cultures were detected using the Duolink *In situ* Orange Starter kit Goat/Rabbit (Sigma Aldrich).

Intracellular cAMP assay

Cells were treated with 0.5 μM IBMX prior to any agonist treatments as indicated. For Figure 1 whole cell cAMP was measured via competitive immunoassay (Assay Designs) as previously described (Hanyaloglu et al., 2005). For all other figures cells were washed in cold PBS and lysed in Cisbio LB1 lysis buffer LB1 (64KL1FDF) with 0.2% Triton X-100 and centrifuged for 15 min at 16,000g. Lysates were normalized to protein concentration determined by Coomassie (Bradford) Protein Assay Kit and analyzed as per manufacturers protocol (Cisbio Gαs dynamic assay) using a PHERAstar FSX.

Bioluminescence resonance energy transfer (BRET)

At 85–90% confluence primary myometrial or HEK 293 cells were transfected with 1μg EP2-Rluc and increasing amounts of OTR-Venus (0.01–2 μg) using Lipofectamine 2000 (Invitrogen) in DMEM containing 10% fetal bovine serum without antibiotics. 48h post-transfection cells were manually lifted and resuspended in 450 μL PBS containing 10% fetal bovine serum. 50μL of each condition was seeded into wells of a white-bottomed 96 well plate. Coelenterazine-h (5μM; Promega) was added and emission was measured at 488nm and 530nm using a FLUOstar spectrofluorometer. Venus YFP was measured via excitation at 485nm and emission at 540nm.

Western Blot

Confluent myometrial cells were lysed in RIPA buffer containing 1% Triton X-100, 1% Sodium deoxycholate, 0.1% SDS, 150mM NaCl, 10mM Tris, 1mM EDTA plus 1mM sodium orthovanadate and one Pierce EDTA-Free protease inhibitor mini tablet. 20–40μg of lysate as determined via Bradford assay plus Laemmli buffer (0.5M pH 6.8 Tris, 10% SDS, 1% Bromophenol blue, 2% B-ME, 20% Glycerol) were separated with 1X SDS running buffer at 140V on SDS-PAGE gels polymerized with TEMED and ammonium persulfate: separating gel (40% poly, 1M pH 8.8 Tris, 10% SDS), resolving gel (40% poly, 1M Tris (pH 6.8), 10% SDS). Proteins were transferred onto nitrocellulose membranes before blocking in 5% milk TBS-T for 30 min and incubated overnight at 4°C with primary antibody and blocking buffer (1:1000). Membranes are then incubated in TBS-T with appropriate secondary antibody for 1-3h (1:2000) before signal detection using HRP substrate and chemiluminescent imager (ImageQuant LAS 4000).

Measurement of secreted PGE2 and IL-6

1mL media was collected from cultured myocytes stimulated with/without agonists. Quantification of PGE2 (Enzo Lifesciences) or IL-6 (Biolegend) concentrations was determined via ELISA as per manufacturer's instructions.

QUANTIFICATION AND STATISTICAL ANALYSIS

All statistical data analyses were performed in GraphPad Prism 8. In general, two-tailed, unpaired *t* test was performed to determine statistical significance. In cases of multiple comparison one-way ANOVA was used with either Tukey's post-hoc test when comparing every mean with every other mean, Sidak's post-hoc test when comparing a select set of means, or Dunnett's method when comparing every mean with a control mean. In cases where patient data had been normalized to basal or agonist response one-sample t test was performed to allow comparison against a bounded value. For each test, $p < 0.05$ was considered significant. Specific tests are noted in each legend.

Cell Reports, Volume *40*

Supplemental information

Functional rewiring of G protein-coupled receptor signaling in human labor

Abigail R. Walker, Camilla B. Larsen, Samit Kundu, Christina Stavrinidis, Sung Hye Kim, Asuka Inoue, David F. Woodward, Yun S. Lee, Roberta Migale, David A. MacIntyre, Vasso Terzidou, Francesca Fanelli, Shirin Khanjani, Phillip R. Bennett, and Aylin C. Hanyaloglu

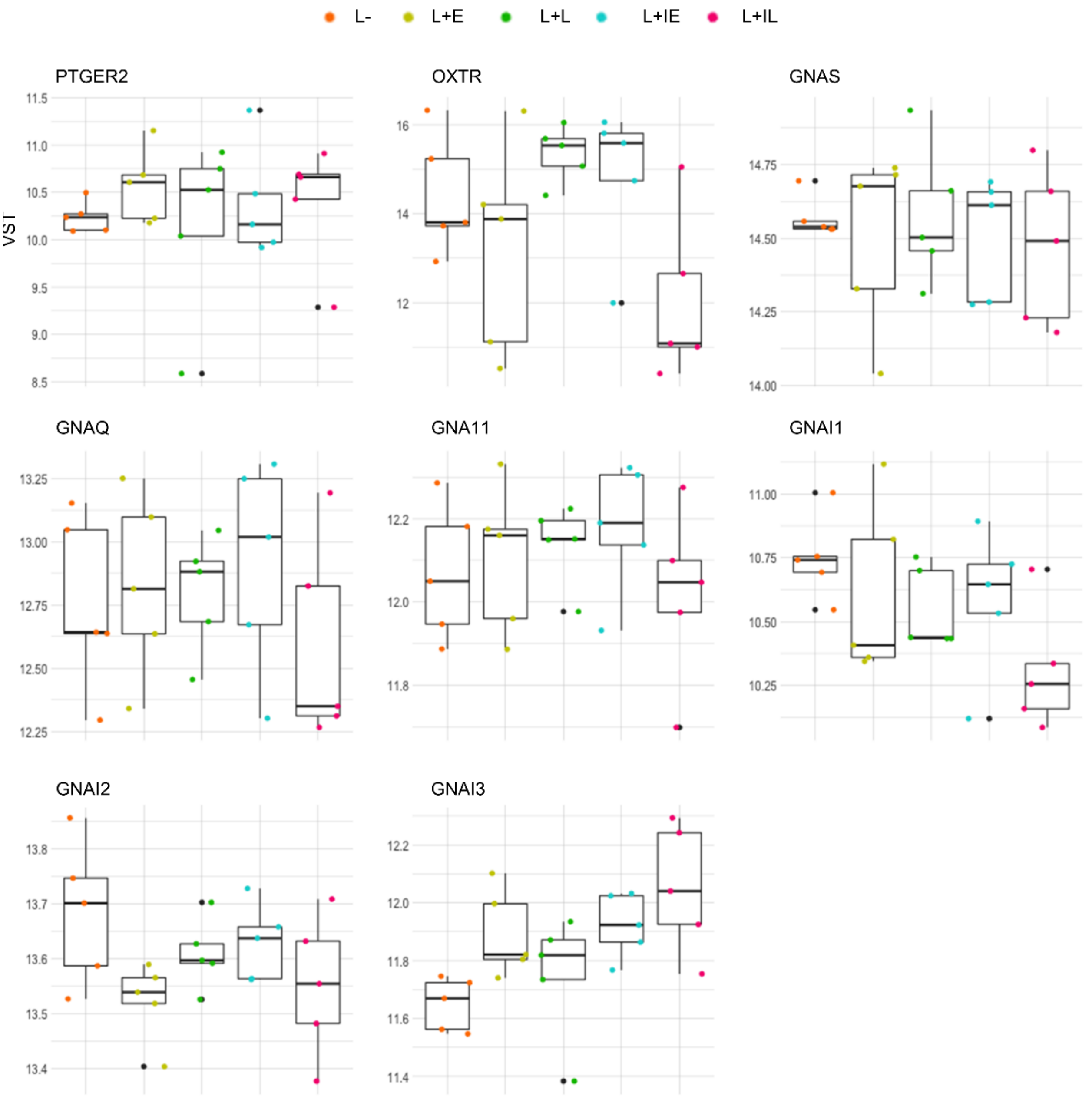

Figure S1. Expression of EP2, OTR and their Ga subunits in myometrial cells during labor, related to Figure 1. Expression levels of EP2 (Ptger2), OTR (Oxtr) and Gα subunits associated with EP2 and OTR signaling, GNAS, GNAI1/I2/I3/O and GNAQ/11, in term pregnant myometrium via RNA-Seq of patient samples used for functional studies in Fig. 1A-C. L- = non-laboring, L+E = early-stage spontaneous labour, L+L= late-stage spontaneous labour, L+IE =

early-stage induced labour, L+IL= late-stage induced labor. Early labor refers to cervical dilation < 3 cm, late labour refers to cervical dilation > 3 cm. Each circle represents one patient with outliers indicated with black circle. VST = variance stabilizing transformation. n=5.

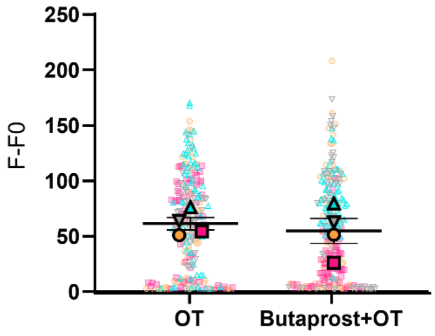

Figure S2. Pre-treatment of myocytes with butaprost does not affect OT-induced intracellular Ca^{2+} responses, related to Figure 2. Intracellular Ca^{2+} release in cultured term pregnant myocytes. Cells were imaged live before and following stimulation with butaprost (10 µM), with or without 1h OT pretreatment (100 nM). Data is the maximal fluorescent intensity normalized to unstimulated baseline (F-F0) shown for each cell analyzed, overlaid with the mean of the maximum cell intensity per experiment ± SEM. Each cell analyzed is represented and color coded for each biological repeat. Data shown as mean intensity ± SEM. 30 cells were imaged in duplicate per sample, n=4.

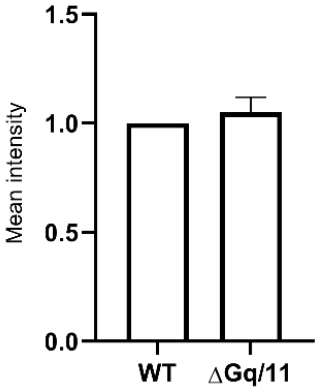

Figure S3. Cell surface expression of FLAG-tagged EP2 in ΔGαq/11 versus wildtype (WT) HEK 293 cells, related to Figure 4. The cell surface levels of human FLAG-tagged EP2 transiently expressed in either HEK 293 cells lacking GNAQ and GNA11 (ΔGαq/11) or WT HEK 293 cell was analyzed by anti-FLAG antibody staining in live cells and quantitated by flow cytometry. Mean fluorescence intensity is normalized to WT expression. Data are mean ± SEM of n=3.

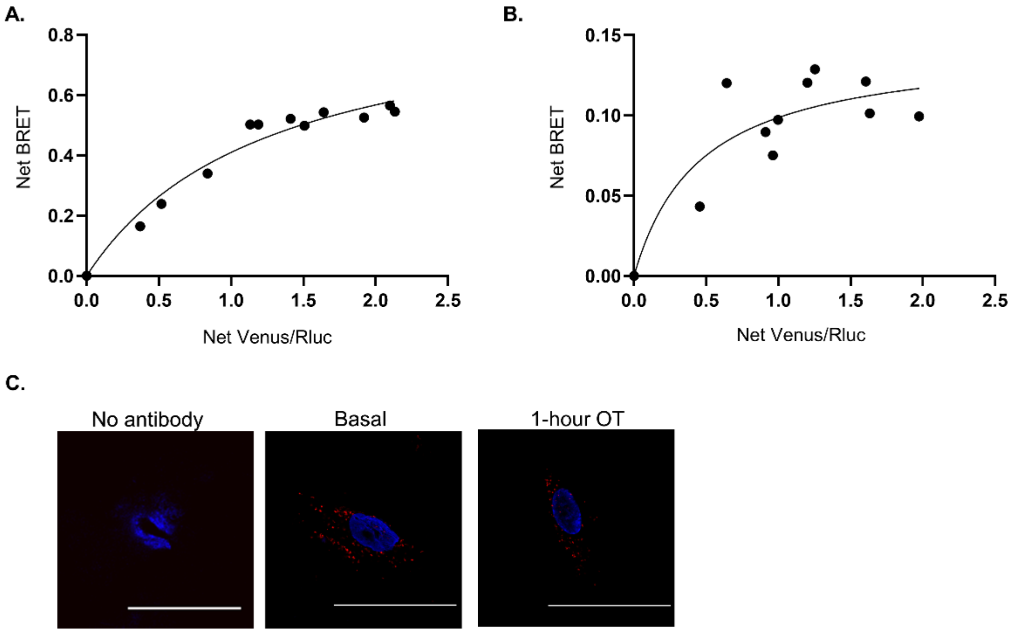

Figure S4. EP2-OTR heteromers are pre-formed in both HEK cells and primary human term pregnant myometrial cells, related to Figure 5. (A-B) Bioluminescence resonance energy transfer (BRET) saturation curves in (A) HEK 293 cells and (B) primary non-laboring myometrial cells, transiently transfected with EP2-RLuc8 and increasing levels of OTR-Venus. Coelenterazine was added at a final concentration of 5 µM. Emission was measured at 488nm and 530nm. Venus YFP was measured via excitation at 485nm and emission at 540nm. Representative curve shown, n=3. **(C)** Proximity ligation assay (PLA) with and without OT treatment (100 nM, 1 h) in primary myocytes to detect EP2/OTR heteromers. Red spots indicate positive PLA signal. DAPI for nuclear staining is in blue. Representative images from ~20 cells imaged across 2 patient samples. Scale bar = 50 µm.

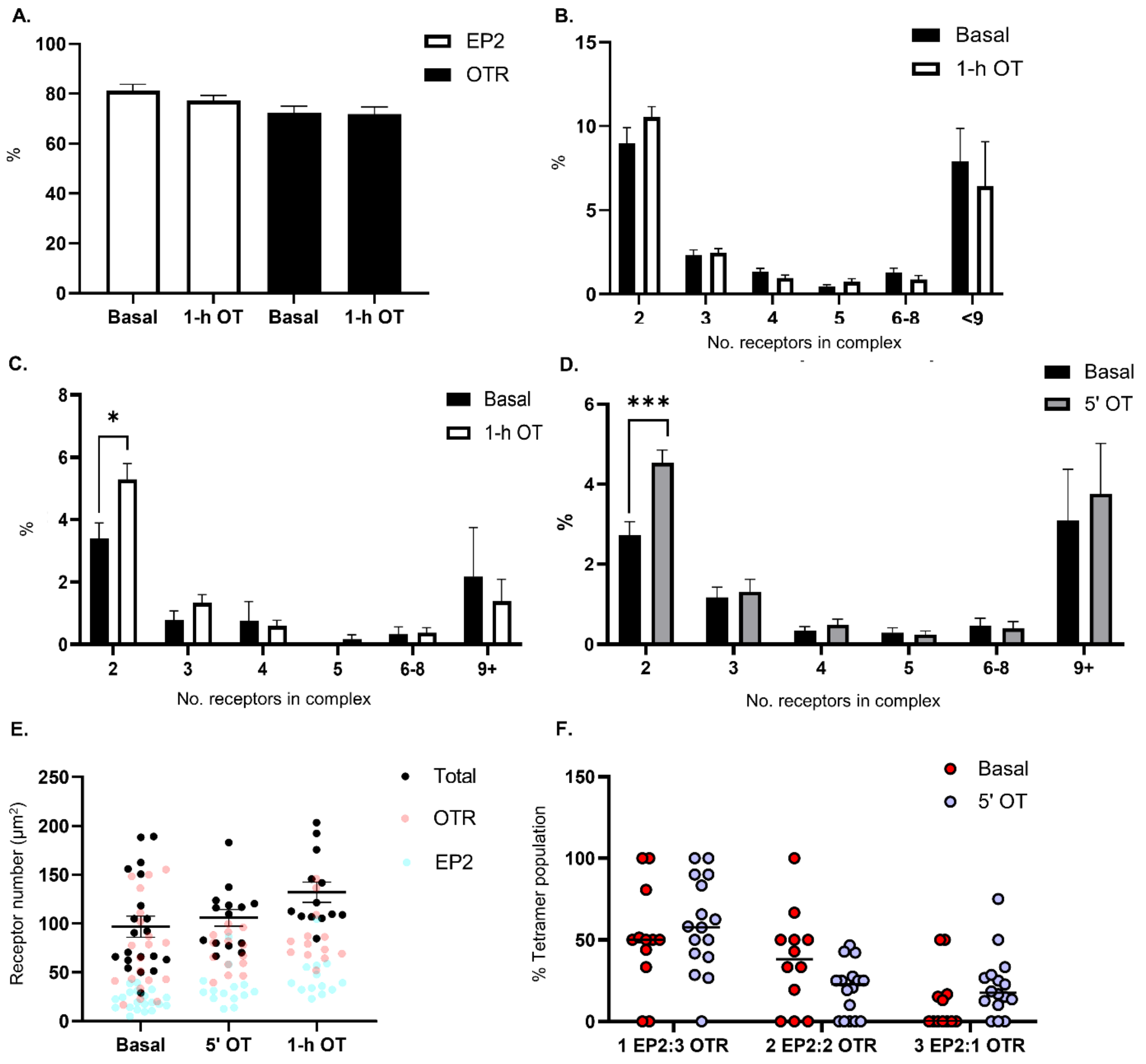

Figure S5. The EP2 and OTR cell surface monomer and homomer populations, related to Figure 5. PD-PALM analysis of cell surface EP2/OTR monomers and homomers in HEK 293 cells co-expressing FLAG-EP2 and HA-OTR with/without 1h OT stimulation (100 nM). EP2 and OTR monomer **(A)** and both OTR homomer (**B**), and EP2 homomer (**C**) populations were quantified from the same experiments presented in Fig. 5B as percentage of total EP2 or OTR, respectively, with/without 1h OT stimulation (100 nM). Data are mean ± SEM. n=4 independent experiments of 4-6 cells per experiment. Data represented as mean ± SEM. Unpaired *t* test: $p<0.05$.

(D) EP2-OTR heteromers with/without OT (100 nM, 5-minutes) expressed as % of all receptors, stimulated and imaged alongside cells presented in Fig. 5B. Mean ± SEM, n=4 independent experiments of 4-6 cells each. **(E)** Receptor number across PALM experiments from Fig. 5B and Supplementary Fig. S5D. **(E)** Composition of heterotetramers stimulated with/without OT (5-minute). Mean ± SEM, n=7, 2-6 cells each. *p< 0.05, ***P < 0.001, unpaired, two-tailed Student's *t*-test.

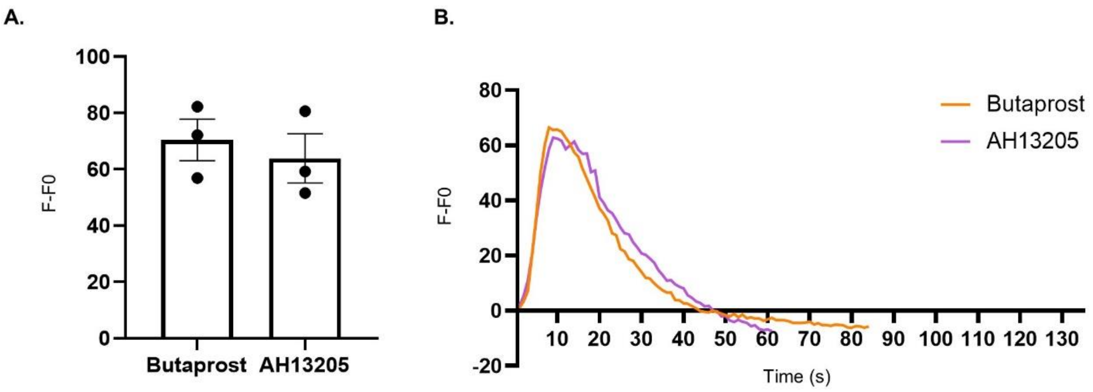

Figure S6. Butaprost and AH13205 induce Ca^{2+} release in non-laboring myocytes, related to Figure 6. Intracellular Ca^{2+} release in myocytes cultured from term pregnant non-laboring myometrium. Cells were imaged before and following stimulation with either AH13205 (10 µM) or butaprost (10 µM). **(A)** Data is the mean of the maximum fluorescent intensity of 30 cells per experiment normalized to unstimulated baseline (F- F0), shown as mean ± SEM. 30 cells were imaged in duplicate per sample, n=3.. **(B)** Representative fluorescent intensity traces over time from Fig. S6A.

Table S1. Accession numbers for RNAseq, Related to Figure 1.

Accession number	Labour type	Depository
ERR9539548	Early Induced	ENA
ERR9539547	Late	ENA
ERR9539546	Late Induced	ENA
ERR9539545	Late Induced	ENA
ERR9539544	Late Induced	ENA
ERR9539543	Late Induced	ENA
ERR9539542	Late Induced	ENA
ERR9539541	Late	ENA
ERR9539540	Late	ENA
ERR9539539	Late	ENA
ERR9539538	Late	ENA
ERR9539537	Early Induced	ENA
ERR9539536	Early Induced	ENA
ERR9539535	Early Induced	ENA
ERR9539534	Early Induced	ENA
ERR9539533	Early	ENA
ERR9539532	Early	ENA
ERR9539531	Early	ENA
ERR9539530	Early	ENA
ERR9539529	Early	ENA
ERR9539528	Non-laboring	ENA
ERR9539527	Non-laboring	ENA
ERR9539526	Non-laboring	ENA
ERR9539525	Non-laboring	ENA
ERR9539524	Non-laboring	ENA
